# Depth is a strong driver of functional diversity in Caribbean reef-fish communities

**DOI:** 10.1007/s00442-026-05902-9

**Published:** 2026-05-15

**Authors:** Juliette Jacquemont, Simon J. Brandl, Carole C. Baldwin, Luke Tornabene

**Affiliations:** 1 School of Aquatic and Fishery Sciences, University of Washington, 1122 NE Boat St, Seattle, WA 98195 USA; 2 National Center for Ecological Analysis and Synthesis, University of California, 1021 Anacapa St Suite 300, Santa Barbara, CA 93101 USA; 3 Department of Marine Science, Marine Science Institute, The University of Texas, 750 Channel View Dr, Port Aransas, Austin, TX 78373 USA; 4 Department of Vertebrate Zoology, National Museum of Natural History, Smithsonian Institution, Washington, DC USA

**Keywords:** Coral reef, Reef fish, Mesophotic, Functional traits, Deep reef

## Abstract

Functional diversity patterns have been thoroughly described for reef fishes across geographic gradients, but the effect of depth on these patterns remains largely unknown. Using diet, life-history, and behavioral traits, we evaluated the functional diversity of Caribbean reef-fishes from the surface to 450 m depth to test if taxonomically distinct communities along the reef slope are characterized by similar or distinct ecological traits. Core ecological traits found in shallow reef-fish communities were conserved in the upper section of deep reefs (i.e., mesophotic zone) across three taxonomically distinct assemblages. By contrast, the deepest reef communities (i.e., rariphotic zone) displayed a distinct functional structure, mainly driven by trait loss. Functional redundancy was highest in the deepest reef communities and was generated by intrageneric diversification and functional convergence across phylogenetic lineages. The decrease in functional richness and divergence combined with an increase in functional redundancy suggest that environmental filtering is the dominant evolutionary constraint shaping the functional diversity of deep-reef fishes.

**Supplementary Information:**

The online version contains supplementary material available at 10.1007/s00442-026-05902-9.

## Introduction

Trait-based ecology, which characterizes communities through measurable species attributes, has been key to elucidate community assembly rules, mechanisms driving biodiversity patterns, and linkages between diversity and ecosystem functions (Díaz and Cabido 2001; McGill et al. 2006; Tilman et al. 1997). While trait analyses were initially developed for terrestrial systems, their principles have more recently been transferred to investigate the ecology, biogeography, and vulnerability of marine communities (Butt et al. 2022; Cheung et al. 2005; Mouillot et al. 2014). Trait-based approaches can be particularly useful to illuminate the ecological processes and assembly rules of marine communities that are difficult to access, observe, and for which traditional ecological experiments are logistically challenging. For example, functional diversity analyses based on traits have advanced the understanding of evolutionary and ecological processes at play in the deep sea (Myers et al. 2021), on hydrothermal vents (Alfaro-Lucas et al. 2024), and on deep reefs (Stefanoudis et al. 2023). Yet, the functional ecology of deep ecosystems remains largely undocumented compared to their shallow counterparts. For instance, while dozens of studies have documented the functional ecology of shallow reef communities (Hadj-Hammou et al. 2021), only a handful have focused on deep reefs.

Deep reefs form contiguous extensions of shallow reefs along tropical and subtropical coasts from 40 m down to ca. 300 m and remained until recently largely undocumented due to the technical challenges of accessing these depths (Kahng et al. 2014; Lesser et al. 2018). The increase in deep-reef studies in the past decade has revealed their diversity and taxonomic distinctiveness (Laverick et al. 2018; Rocha et al. 2018) but also the multiple human and environmental pressures threatening these ecosystems (Pinheiro et al. 2019; Rocha et al. 2018), underscoring the urgency to improve their management (Soares et al. 2020). However, because experimental approaches are technically difficult to conduct at depth, deep-reef studies have largely been limited to taxonomic descriptions, with limited understanding of ecosystem functioning (Radice et al. 2024). This knowledge gap is true for all deep-reef taxa including fish, the most diverse vertebrate group of the planet.

In this context, trait-based approaches appear as a valuable and untapped resource to advance our understanding of the ecological and evolutionary processes shaping deep-reef fish communities. Previous studies have established that fish richness and abundance decline with depth across ocean basins, and identified taxonomic breaks defining distinct altiphotic (ca. 0–30 m), mesophotic (ca. 40–200 m), and rariphotic (ca. 200–300 m) fish communities (Baldwin et al. 2018; Jacquemont et al. 2024a; Stefanoudis et al. 2023; Weijerman et al. 2019). However, apart from the repeated observation that herbivorous fishes decline with depth (Bejarano et al. 2014; Coleman et al. 2018), taxonomic breaks occurring along the reef slope have not been correlated with functional shifts. The few studies that have characterized the functional diversity of deep-reef fishes have found limited functional changes between altiphotic and mesophotic communities, mostly driven by variations in relative trait abundance and by shifts in predominant trophic guilds (Bosch et al. 2023; Loiseau et al. 2023; Medeiros et al. 2021). Only two studies (Stefanoudis et al. 2023, 2025), both performed in the Indian Ocean, have investigated the functional ecology of reef fishes occurring below 150 m (i.e., rariphotic communities), and a single study has documented the functional ecology of Caribbean mesophotic fishes (Pinheiro et al. 2023a). While the prevailing hypothesis is that environmental filtering, i.e., the selection of specific traits within a community due to environmental constraints, is the dominant evolutionary driver in extreme ecosystems, trait-based approaches have reached varying conclusions regarding its prevalence on deep reefs depending on the depth range and functional metrics considered (Bridge et al. 2016; Pinheiro et al. 2023a; Stefanoudis et al. 2023). Lastly, many studies have discussed the potential of deep reefs to provide refuge to shallow species from disturbances (Lindfield et al. 2016; Medeiros et al. 2021), but whether deep-reef communities benefit from ecological characteristics associated with resilience, such as high functional redundancy or the prevalence of generalist species, has not been investigated.

Here, we characterize the functional diversity of Caribbean reef fishes across their entire depth range of occurrence (0–450 m) using seven traits related to life-history, behavioral, and trophic ecology. We first assess how trait composition varies across depth within trait categories. We then perform multi-trait analyses to test (1) whether taxonomically distinct fish communities along the reef slope are functionally distinct, and if so, which traits drive functional distinctiveness; (2) whether environmental filtering (denoted by trait loss, decreased functional space, and decreased functional divergence) or biotic competition represents the predominant evolutionary constraint on deep reefs; and (3) whether functional redundancy and evenness increase on deep reefs, which would suggest higher ecological resilience of these communities to disturbances. Lastly, we discuss how ‘effect’ traits could affect ecosystem processes on deep reefs and what ‘response’ traits suggest regarding the main ecological constraints at play for deep-reef fishes.

## Methods

### Data collection

#### Submersible transects

Fish data were collected from direct visual observations by trained ichthyologists using manned-submersible diving. We conducted submersible-diving surveys at four Caribbean islands (Curaçao, Bonaire, St. Eustatius, Roatán) from 10 to 480 m in Roatán, and from 40 to 300 m at the three other sites. Most data collection occurred between 2011 and 2017 as part of the Smithsonian Deep Reef Observation Project (DROP) which featured > 100 submersible dives. A minimum of 11 submersible dives (each 3–6 h long) were performed at each island (Table S1), during which descending (first half of dives) and ascending (second half of dives) transects were conducted on two different trajectories along the reef slope. Submersible observations were coupled with cryptobenthic fish sampling using anesthetic across depth. See Baldwin et al. (2018) and Robertson et al. (2022) for additional details on submersible surveying methods.

Additional submersible-diving surveys were conducted in Curaçao in August 2024 because rarefaction curves indicated that previous sampling efforts had not fully captured deep-reef fish diversity (Jacquemont et al. 2024a). One additional horizontal transect per depth strata, approximately 120 m long was conducted at 45, 60, 80, 100, 115, 130, 150, 175, 200, 225, and 250 m to enrich previous datasets.

#### SCUBA transects

We acquired shallow fish observations in Bonaire (August 2024) and in Curaçao (December 2023) to complement submersible observations that started at 40 m at these locations. Underwater visual censuses were conducted using SCUBA diving by two trained observers (JJ and LT) along 2 m wide x 30 m long transects at 5, 10, 20, and 30 m depth. Fish observations were limited to individuals occurring within 2 m of the seafloor to match submersible observations, which do not capture individuals occurring midwater or at the surface. Each observer conducted the same number of transects at each depth to avoid unbalanced observer bias. Five to six replicates were performed for each depth and at each island, spaced out by at least 50 m. Survey locations were chosen to match previous submersible survey areas, i.e., east of Willemstad in Curaçao and between Bachelor’s Beach and Angel City in Bonaire. We normalized abundances by transect length, which varied in Curacao due to bottom-time constraints and challenging current conditions (Table S1).

Given differences in sampling method employed (SCUBA-diving vs. manned submersible) and the difficulty to quantify the exact sampling effort performed by submersible diving at each depth strata, we computed species and functional entity rarefaction curves to identify sampling biases that could affect our results. Rarefaction curves (Fig S16-17) suggested comparable and high levels of species and functional entity sampling.

#### Fish traits

We compiled data on seven traits commonly evaluated in reef-fish functional studies (Loiseau et al. 2023; McLean et al. 2019; Mouillot et al. 2014): maximum body length, diet, position in the water column, diel activity, gregariousness, mobility, and reproduction (see Table S2 for categories considered within each trait categories). These traits describe important aspects of fish life-history, behavior, and trophic ecology that influence and respond to ecosystem processes such as connectivity, productivity, and nutrient cycling (Villéger et al. 2017).

We assigned trait values at the species levels using the Fishes of the Greater Caribbean database [Smithsonian Tropical Research Institute, STRI; Robertson and van Tassell (2015)] to document diets, water column position, reproduction, and size at maturity. We assigned mobility, gregariousness, and diel activity traits using expert knowledge complemented by literature search. When species traits were not available, we used trait data from closely related species when those traits were known to be conserved at the corresponding phylogenetic scale (e.g., members of the genus *Chromis* have similar reproductive modes; members of the family Gobiidae exhibit parental care over eggs).

In some instances, more than one category was assigned to a given species when this best reflected the species’ ecology. For example, *Sphyraena barracuda* was assigned to near-surface, mid-water, and near-bottom categories. The unique combination of trait categories represented by a given species was used to define a single functional entity per species, which was then used to compute functional trait spaces. To do so, we spread trait variables into as many columns as individual categories for that trait, and used a binary code to indicate the presence or absence of trait categories in a given species (Data S1). The unique sequence of binary values was then used to define a single functional entity per species. As a result, although some species were assigned to multiple trait categories, each species was only represented once in functional analyses.

### Data analysis

All analyses were performed using R version 4.4.1 (R Core Team, 2024) in R Studio.

#### Variations in individual traits

To assess how the relative prevalence of trait categories varied across depth, we calculated the community weighted mean (CWM*i*) of each trait category at each depth bin and site using the *dbFD* function from the {FD} package. Because we reclassified traits into as many binary variables as individual categories for that trait, CWM*i* represented the proportion of the total community abundance displaying trait *i* and was bounded between 0 and 1. As a result, the sum of CWM*i* across n trait categories is at minima 1, when each species is attributed a single trait category, and at maxima *n*, when all species are attributed all trait categories. We treated traits as non-ordered categories and weighted traits by abundance matrices which were square-root transformed to avoid overemphasizing the importance of rare species while controlling for extremely abundant ones. We tested the effect of depth and location on CWM values of individual trait categories using General Additive Models (GAMs):

$$CWM=\alpha\: + s\left(depth\right) + depth:location$$

with s(x) specified as a smooth non-parametric function and depth: location the interaction between depth and location (i.e., the four islands).

We fitted CWM*i* values using beta-regression functions and an REML method to minimize overfitting. We ran models using the {mgcv} package and visualized fits using the {marginaleffects} package.

#### Multidimensional trait space

To compare the breadth of ecological traits in different fish communities down the reef slope, we computed a multidimensional trait space using the six categorical traits for which we had complete data: size, diet, reproduction, position in the water column, gregariousness, and mobility. We excluded diel activity as data gaps prevented the categorization of most deep-reef species. We reclassified traits into as many binary variables as individual categories for that trait (e.g., “position in water column” was reclassified into four binary traits). This allowed us to assign multiple trait categories to a single species when this best reflected its ecology or behavior. To avoid artificially increasing the weight given to traits with more categories, we weighted each category by the inverse of the number of categories within each trait. We computed Gower dissimilarity matrices from these binary variables for each site and depth bin, and projected them in an Euclidean space using the Cailliez correction method (Cailliez 1983). We then performed Principal Coordinates analysis (PCoA) on corrected species-species distance matrices. We calculated the associated convex hull volume based on the two first PCoA axes and plotted species in this two-dimensional space according to their traits. To compare trait spaces across depth zones, we represented the trait space of six taxonomically distinct communities across depths: altiphotic, upper mesophotic, lower mesophotic, upper rariphotic, lower rariphotic, and below the rariphotic. We assigned site-specific depth limits for each of these depth zones based on previous work conducted on these communities by Jacquemont et al. (2024a). Lastly, to assess how trait space varied between fishes from different depth affinity groups, we represented the trait space occupied by species that predominantly occur in the altiphotic/mesophotic, mesophotic, mesophotic/rariphotic, rariphotic, or in the deep sea (below 300 m). We assigned depth affinities to species as in Jacquemont et al. (2024a), where the depth range capturing 75% of a species’ abundance defined its depth affinity. For example, a species whose depth range extended from the altiphotic to the rariphotic zone, but with 75% of its abundance in the mesophotic zone, was coded as having a mesophotic depth affinity. By contrast, if 75% of a species’ abundance straddled two depth zones (e.g., altiphotic and mesophotic), it was coded as having an altiphotic-mesophotic depth affinity.

#### Functional diversity metrics

We calculated functional richness, functional evenness, and functional dispersion using the {FD} package developed by Laliberté and Legendre (2010). These metrics measure complementary and independent facets of trait diversity (Legras et al. 2018). Functional richness (FRic) measures the total space occupied by functional traits along the convex hull volume (Villéger et al. 2008). This metric does not account for relative abundance and is sensitive to trait outliers. Functional evenness and dispersion account for relative species abundance, are independent from species richness, and are much less sensitive to outliers. Functional evenness (FEve) measures the regularity of species abundance along the minimum spanning tree linking all species in the multidimensional trait space (Villéger et al. 2008). This metric is independent of the total functional volume. Functional dispersion (FDis) measures the spread of species across the trait space by calculating the mean distance of species to the center of gravity in the functional space (Laliberté and Legendre 2010) and, as such, accounts for total functional volume.

Finally, we calculated functional redundancy following Ricotta et al. (2016) as the difference between taxonomic diversity (Simpson’s index) and trait diversity (Rao’s quadratic entropy) using the {SYNCSA} package. This approach accounts for species richness and species abundance in its measure of redundancy, reflecting that buffering capacity is achieved through both high numbers of species and high numbers of individuals supporting an ecological role. We used GAMs with loess-smoothers to measure the amount of variance of functional diversity metrics explained by depth.

#### Hierarchical clustering

To examine trait similarity between fish communities down the reef slope and identify trait breaks across depths, we conducted hierarchical clustering analyses using trait composition instead of species composition. We calculated the frequency of trait categories among all individuals found at each 10-m depth bins. This created communities composed of *n* = 32 traits categories as opposed to *n* = 168 trait combinations, which would have been more closely correlated to the underlying species composition (*n* = 254). Using these communities of traits, we calculated the Bray–Curtis dissimilarity matrix, which accounts for trait presence and relative abundance at each depth bin, using a Ward linkage (which forms clusters by minimizing variance within clusters), Bray–Curtis distances, and alpha-value = 10^−7^. Analyses were performed with the {vegan} package. Next, we represented trait similarity between depth bins using a hierarchical cluster dendrogram where depth bins most similar in trait composition are closest in branch distance. We tested for significant differences in trait compositions using Analysis of Similarity Profiles [SIMPROF, (Clarke et al. 2008)] functions from the {clustsig} package.

#### Sensitivity analyses

Functional diversity metrics are sensitive to the total number of traits considered and to the number of categories examined for each trait. We re-calculated the total trait space, the trait space represented in each depth zone, and re-ran hierarchical clustering after excluding successively reproductive traits, gregariousness traits, or diet traits (Figs. S13 to S15). We also projected trait spaces using the first and third PCoA axes to test if results were sensitive to PCoA axes retained (Fig. S12).

## Results

**Fig. 1:**
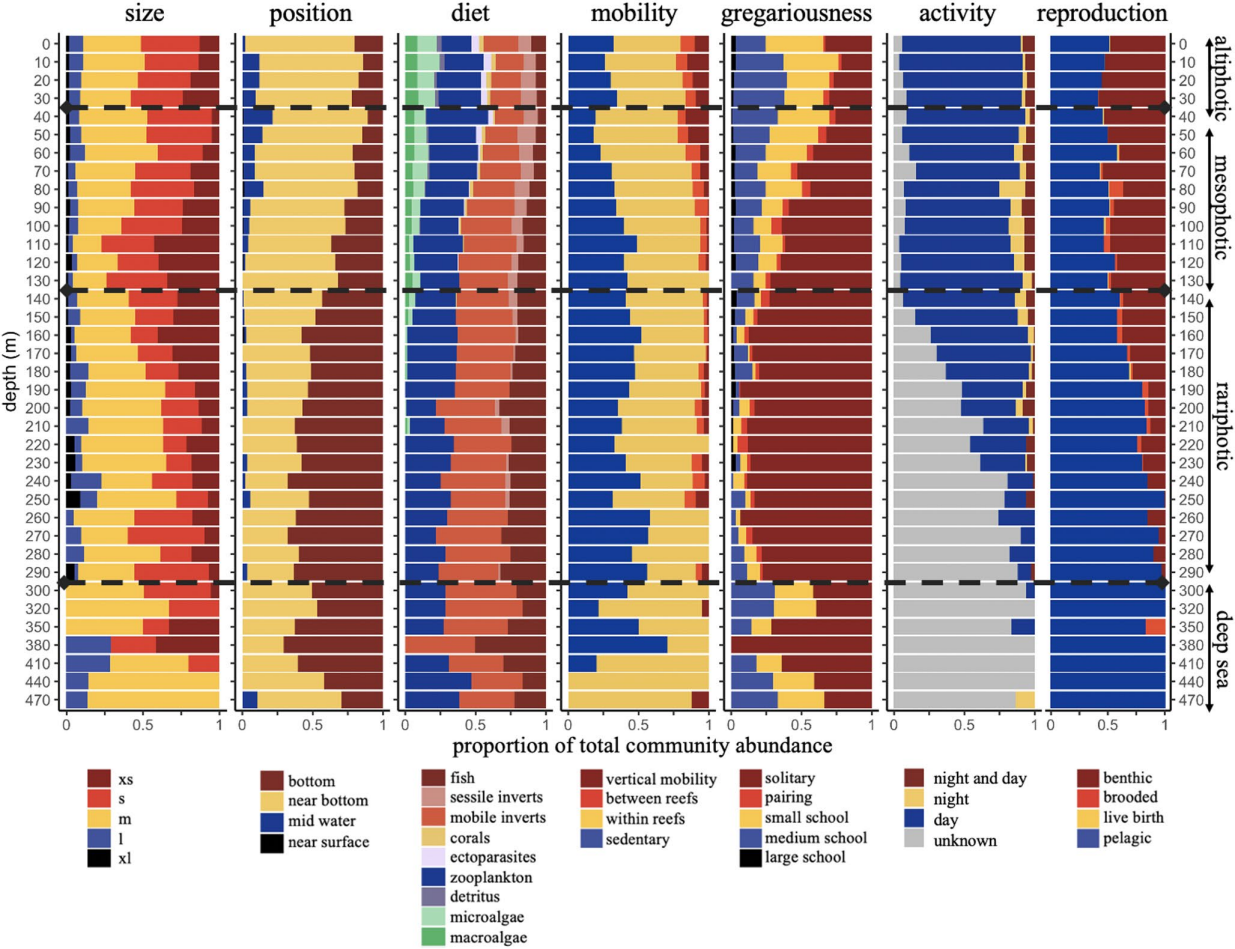
Trait composition across depth. Color fillings indicate different trait categories and bar length represents proportion of total abundance. Black segmented lines indicate the depths of main taxonomic breaks separating distinct fish communities in the Caribbean

We observed 254 fish species between 0 and 450 m depth, representing a total of 168 distinct trait combinations. The relative abundance and the number of species supporting trait categories varied significantly with depth (Fig. 1, Fig. S3). Across all locations, mesophotic and upper rariphotic communities (60–200 m) hosted two to four times more very small-sized fish species (*p* < 0.001) than other depth zones (Fig. 1, Fig. S4) and featured the lowest proportions of large fish. Large and very large species were rare (< 10%) across all depths, but their relative representation increased in the rariphotic and below (> 150 m) due to decreases in abundance of other size classes. The proportion of medium-sized species was relatively stable from shallow to rariphotic reefs but increased below the rariphotic.

Reef communities were dominated (> 80%) across depths by bottom-oriented fish (bottom and near-bottom). The proportion of bottom species increased with depth from 20% on altiphotic reefs to 80% on rariphotic reefs, while the proportion of near-bottom species decreased, and mid-water species disappeared below the mesophotic (*p* < 0.001, Fig. S4). The diversity of trophic strategies decreased from nine to three between altiphotic and rariphotic reefs, with only planktivores, invertivores, and piscivores present below 150 m. The transition from altiphotic to upper mesophotic communities was characterized by an increase in planktivores and a decrease in ectoparasite cleaners, corallivores, and detritivores, which all disappeared in the lower mesophotic (< 120 m). Herbivores (micro- and macroalgae feeders) declined from altiphotic to lower mesophotic reefs (20 to 5%) and disappeared in the rariphotic (*R* = 0.8, *p* < 0.001, Fig. S4). Trophic composition stabilized below 150 m, with piscivores, invertivores, and planktivores found in similar proportions in the rariphotic and below. Reproduction strategies involving benthic and pelagic eggs were evenly present down to the lower mesophotic, below which the proportion of pelagic-eggs strategies increased up to 90% (*R* = 0.78, *p* < 0.001, Fig. S4). Fish displaying brooding represented a minority at all depths but were more common in the mesophotic and upper rariphotic.

Behavioral traits were also affected by depth, with high mobility and schooling behaviors becoming extremely rare on deep reefs. Species displaying between-reef and vertical mobility represented ~ 20% of shallow communities, 5–10% of mesophotic and rariphotic communities, and were absent below the rariphotic (*R* = 0.38, *p* < 0.001). The proportion of solitary species increased from 30% in the altiphotic to 90% in the rariphotic before decreasing again to ~ 30% below the rariphotic. Species forming small and medium schools represented ~ 60% of fish communities in altiphotic reefs and below the rariphotic, but only ~ 40% in the mesophotic and ~ 10% in the rariphotic. Species predominantly active at night were more common on mesophotic than on altiphotic reefs, but trends at deeper depths could not be assessed due to a lack of species data.

**Fig. 2:**
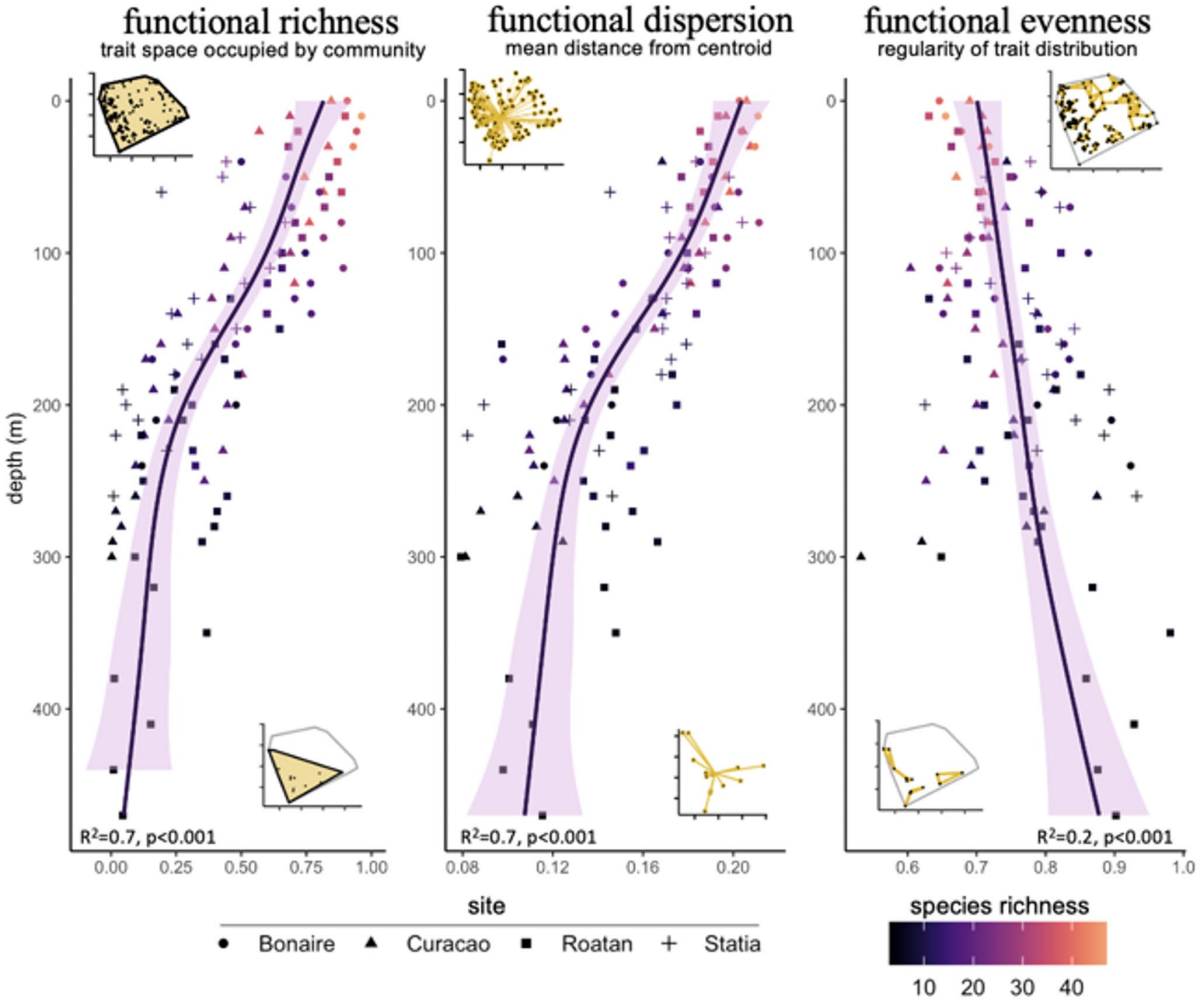
Functional richness, evenness, and dispersion of fish communities across depth. Each dot represents a community from a 10 m depth bin at a given study site. Shapes of dots indicate the study site and color of dots indicate the species richness of the community. Dark lines represent the loess-smoothers fitted to the data and purple shadings indicate the 95% confidence interval associated with the model. Residual standard errors (R^2^) and p-value associated with the loess regressions are indicated in each panel

**Fig. 3:**
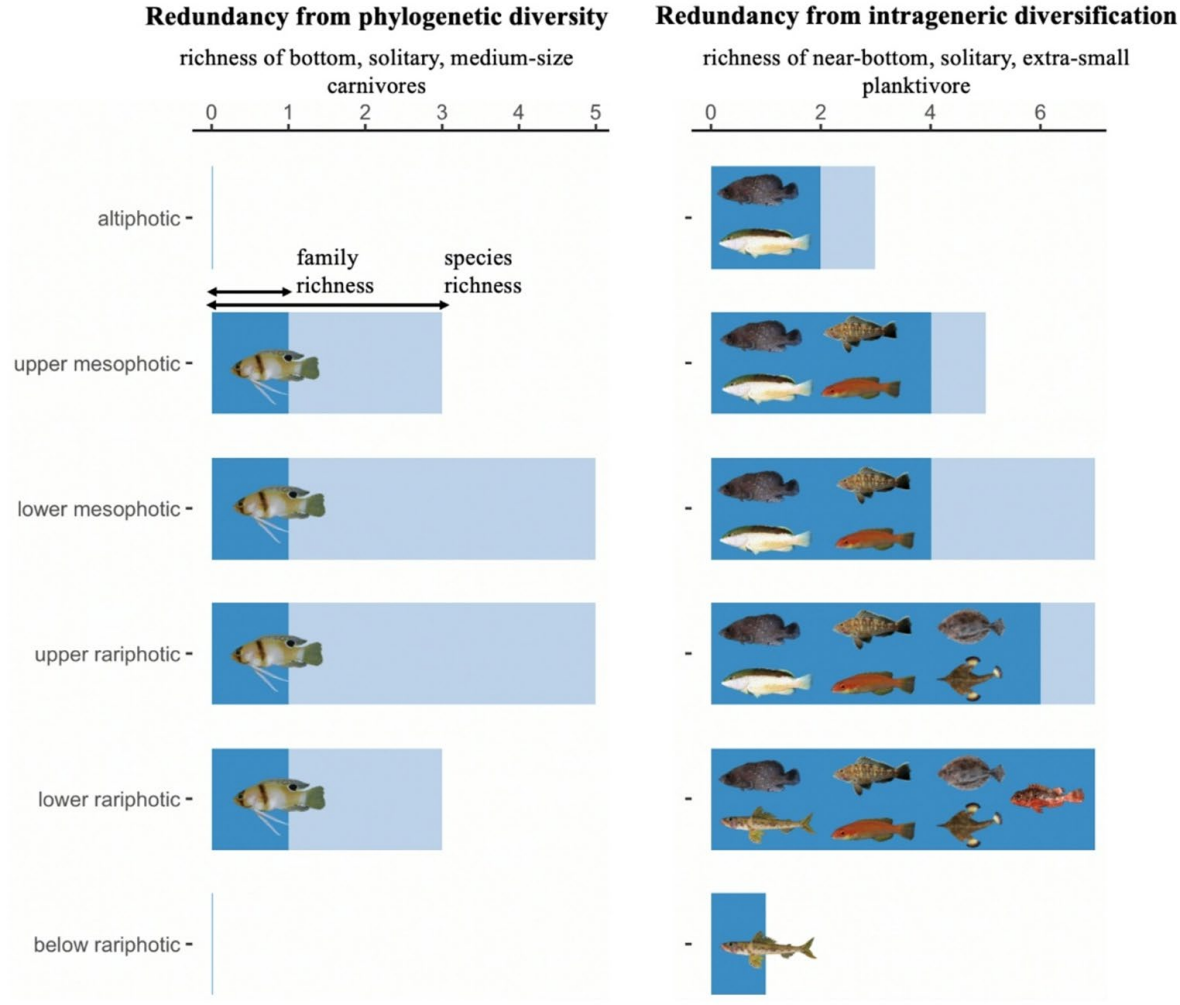
Species and family redundancy supporting two ecological roles on reefs across depth. The height of dark blue bars denotes family redundancy and light blue bars species redundancy. Each family is represented in the figure by an image of one of its members

Functional richness (total functional space occupied) and dispersion (mean distance to centroid) decreased with depth (*p* < 0.001, R^2^ = 0.6) while functional evenness (mean distance between neighbor traits) increased weakly (*p* = 0.001, R^2^ = 0.1, Fig. 2). Richness and dispersion decreased moderately between altiphotic and mesophotic communities, steeply between lower mesophotic and upper rariphotic communities, and reached close-to-null values below the rariphotic. These patterns were mostly consistent across the four studied sites (Fig. S1). The most species-rich trait combination was medium-sized, solitary, bottom predators, which was represented by 11 species, nine genera, and seven families (Fig. 3). The second most species-rich trait combination (near-bottom, solitary, extra-small planktivores) was represented by 9 species from the *Liopropoma* genus. Both trait combinations peaked in redundancy in the upper rariphotic (Fig. 3), which reflected the overall trend of maximum trait redundancy reached in the lower mesophotic and upper rariphotic (*R* = 0.3, *p* < 0.001, Fig. S5).

**Fig. 4:**
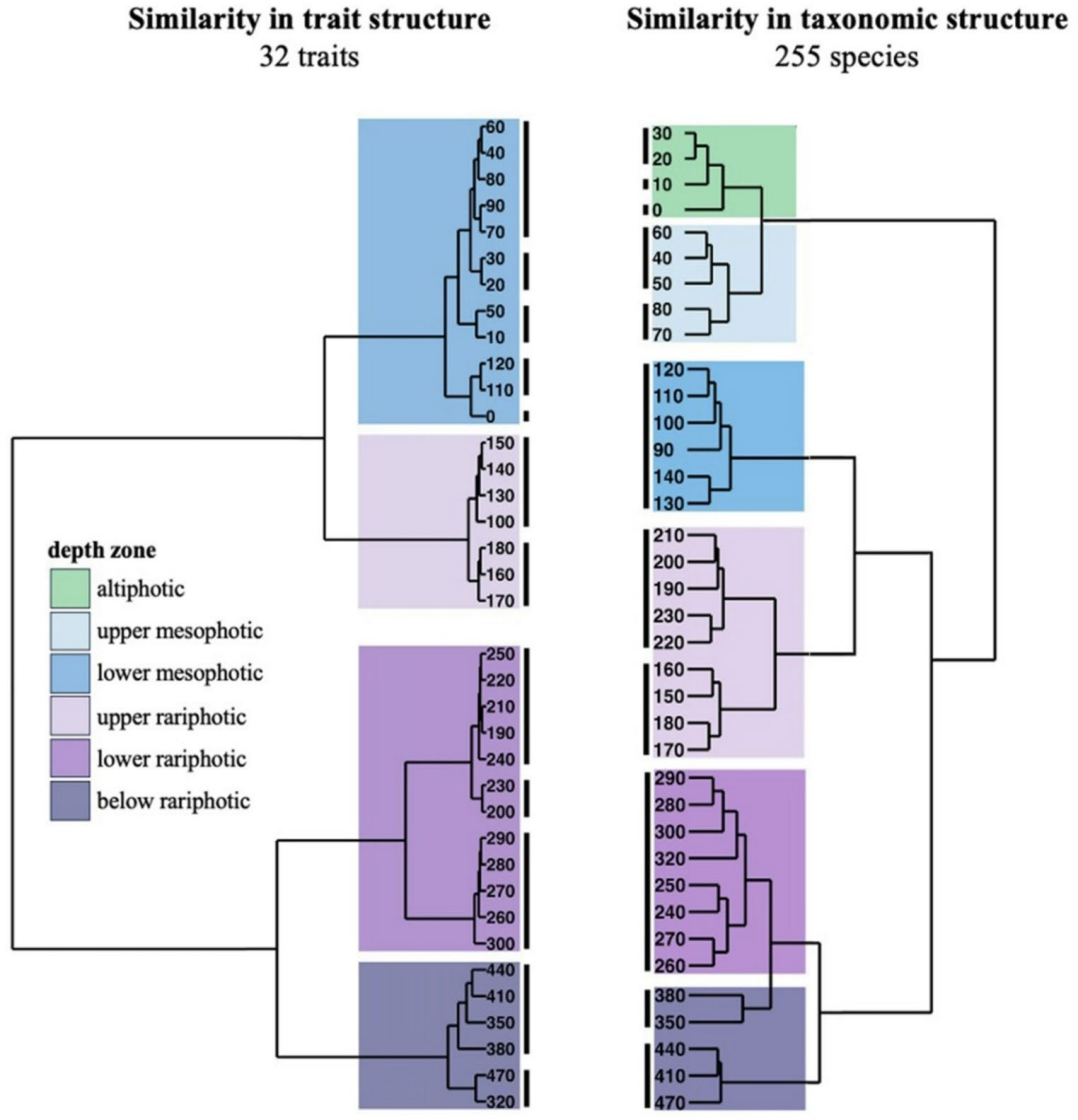
Dissimilarity analyses of reef fish communities across depths based on trait structure (left dendrogram) and on taxonomic structure (right dendrogram). Length of branches in the dendrogram is commensurate to the dissimilarity between depth bins based on Bray–Curtis distance. Significant clusters (SIMPROF analyses, Ward linkage) are indicated by thick black vertical lines. Underlying coloring indicates the depth zone of clusters

We assessed trait similarity between fish assemblages observed at every 10-m depth bin using similarity profile analyses (SIMPROF) and hierarchical clustering. Both depth and location influenced trait composition (PERMANOVA, R2 = 0.54, df = 7, F = 65.8, *p* = 0.001) but trait clusters were primarily divided by depth (Fig. S18). We found 12 distinct clusters of traits across depths, separated into four main branching events (Fig. 4). The main branching in functional structure occurred at 200 m, separating communities above and below the upper rariphotic. A secondary branching indicated functional shifts (i) between lower mesophotic and upper rariphotic communities (~ 130 m) and (ii) between lower rariphotic and below rariphotic communities (~ 300 m). These four main branching events were robust to sensitivity analyses which consisted in removing individual traits from the analyses (Fig. S13 to S15). Shallow to mesophotic communities separated into five functional clusters but unlike taxonomic clusters, distance between functional clusters was small and clusters did not pool consecutive depth bins, which could reflect variability in the vertical limits of depth strata between sites (Fig. S18). By contrast, the depths of deeper functional shifts closely matched those of taxonomic shifts (Fig. 4).

**Fig. 5:**
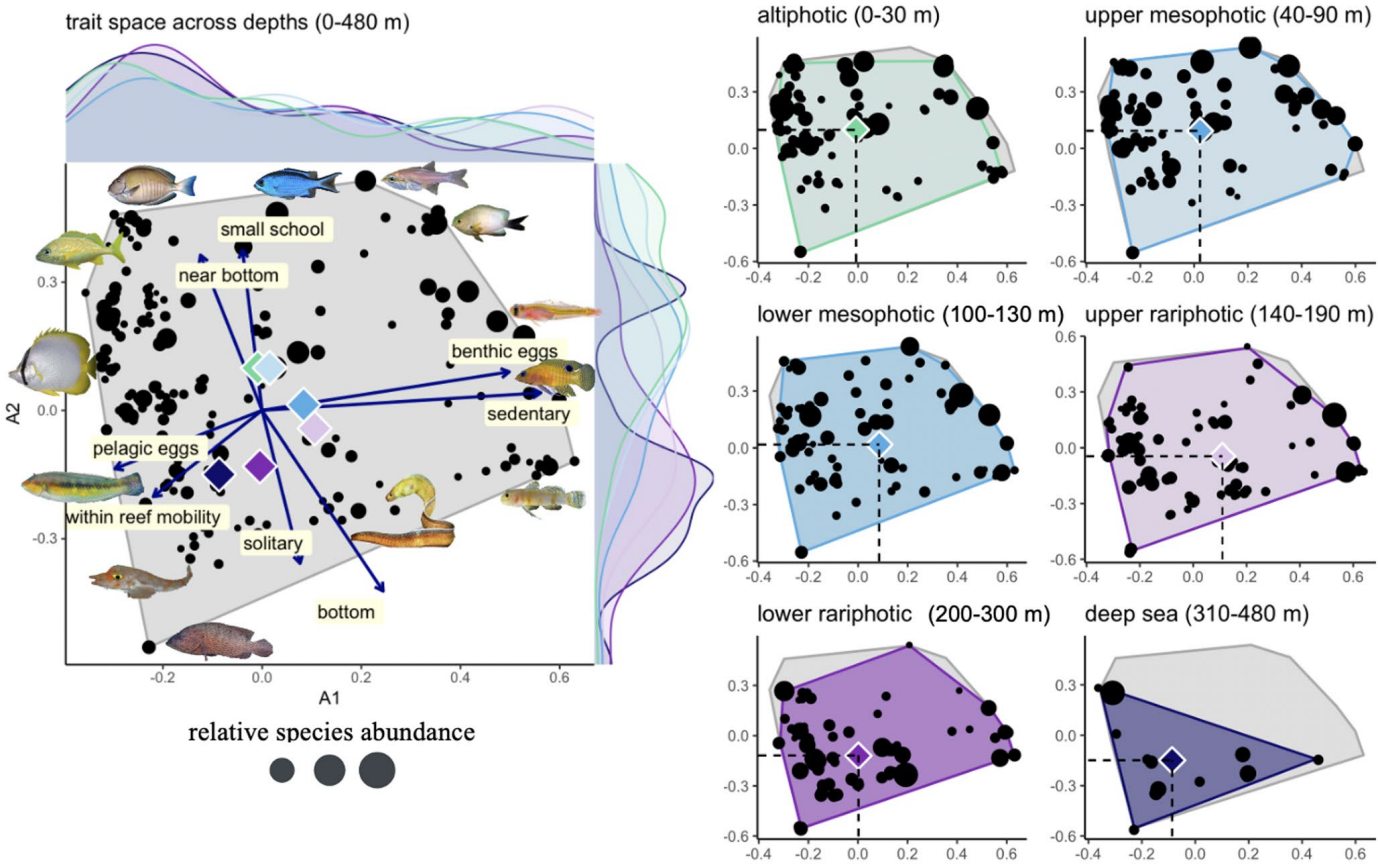
Trait space occupied by fish communities across depths (left plot) and by communities from individual depth zones (right plots) along the first two Principal Coordinate Analyses (PCoA) axes. Black points represent species’ position in the trait space and size of points represents their relative abundance. Colored diamonds indicate the trait centroid for each depth zone. Vectors (left plot) represent the correlation of traits to the PCoA axes. A complete labeling of species occupying the trait space of each depth zone is available in Fig. S6 to S11

Next, we plotted the trait space occupied by reef-fish communities occurring in different depth zones to test if these taxonomically distinct communities occupy distinct trait spaces (Fig. 5). The two first Principal Coordinates Analysis (PCoA) axes captured 18.3% of trait variation. The first axis (10.6% variance) was correlated to reproduction (benthic vs. pelagic eggs) and mobility (sedentary vs. reef mobility) while the second axis (7.7% variance) was correlated to gregariousness (solitary vs. schooling) and position in the water column (bottom vs. near-bottom). Fish communities from altiphotic to rariphotic depths occupied close to the entire trait space with only a small reduction in trait space with depth, indicating that extreme trait combinations were conserved across the entire reef slope (Fig. 5). By contrast, the trait space of communities below the rariphotic shrunk to around half of the total trait space. Similar patterns were found when projecting the trait space along the first and third PCoA axes (Fig. S12). By contrast, the position of communities’ centroids shifted across depth zones. Centroids were ordered by increasing depth along the second PCoA axis, indicating an increase in solitary and bottom species. Centroid of communities increased along the first axis from the altiphotic to the upper rariphotic and decreased in deeper depth zones, indicating a peak in benthic egg and sedentary strategies in the lower mesophotic and upper rariphotic.

**Fig. 6:**
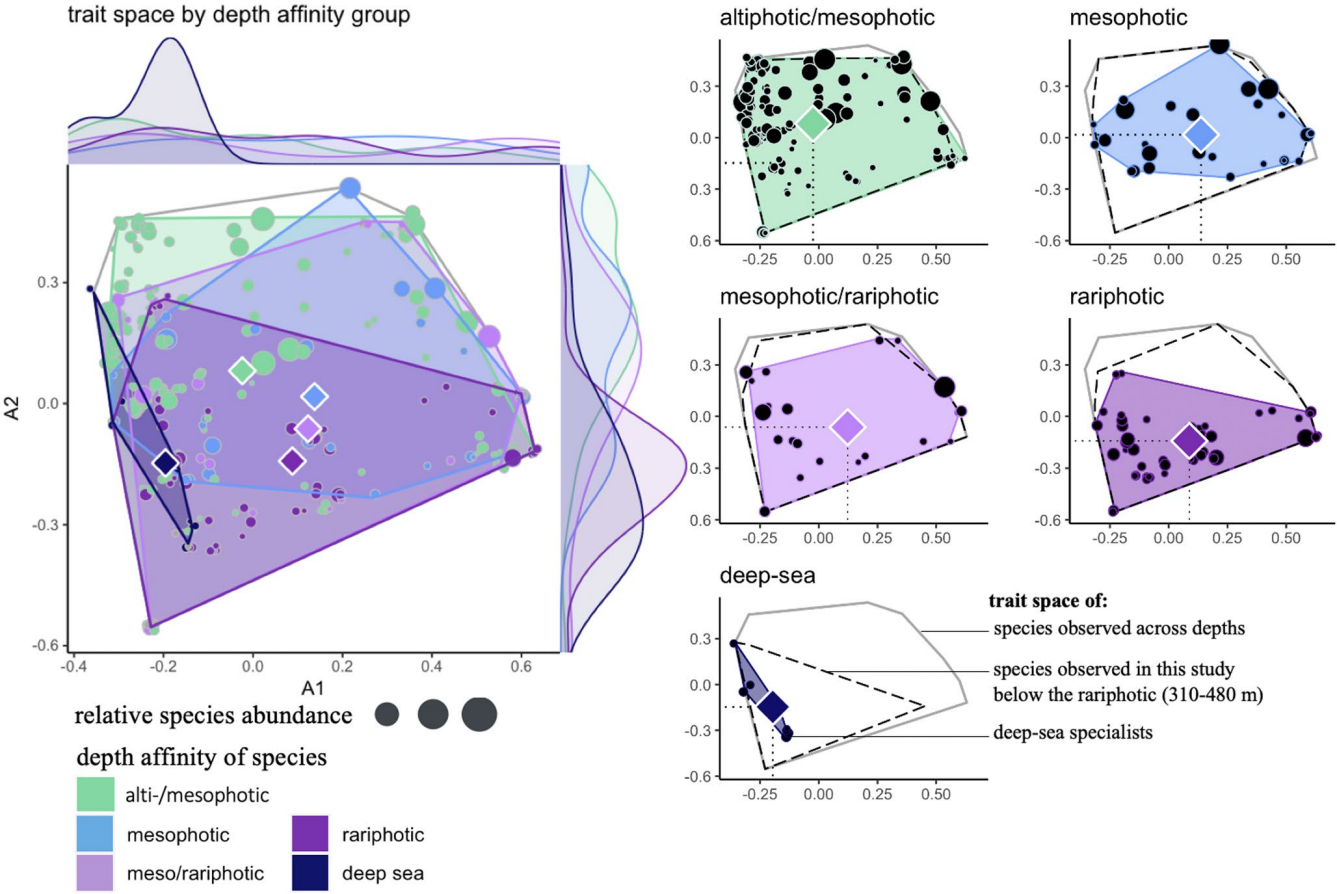
Trait space occupied by fish species across depth affinity groups (left panel) and by individual depth affinity groups (right panels) along the first two Principal Coordinate Analyses axes. Points represent species’ position in the trait space and size of points represents their relative abundance. Colored diamonds indicate the trait centroid of each depth affinity group. The grey outline delineates the functional space occupied by all species, dotted outlines represent the space occupied by communities observed at a given depth zone, and inner colored polygons represent the space occupied by the corresponding depth affinity group

We then examined how trait space varied between depth affinity groups, which categorize species based on their predominant depth range, to test if deep-reef specialists display a more constrained trait space than depth-generalist species. Depth generalists (species spanning altiphotic/mesophotic or mesophotic/rariphotic depths) displayed large and overlapping functional spaces although their centroids indicated distinct mean trait composition (Fig. 6). By contrast, depth specialists (mesophotic, rariphotic, and deep sea) displayed narrower and non-overlapping functional spaces, which was most narrow for deep-sea specialists. The trait space occupied by a given depth affinity group was smaller than the space occupied by the community observed at the associated depth zone, except for altiphotic specialists which occupied the entire functional space of altiphotic communities.

## Discussion

This study examines the trait structure of Caribbean reef-fish communities from the altiphotic to the deep-sea boundary, presenting one of the first functional analyses across the entire depth range of reef-dominated systems. We found that depth is a strong driver of reef-fish trait diversity, with continuous declines in functional richness and divergence, and distinct trait spaces occupied by communities at different depth zones. We found high functional homogeneity across the three shallowest taxonomically distinct fish assemblages, but that functional breaks separate mesophotic, rariphotic, and below-rariphotic communities, with a sharp shrinkage of the trait space below the rariphotic.

### Shifts in trait composition and diversity across depth

The shallowest functional shift separated mesophotic and rariphotic communities, while shallower communities displayed similar combinations of traits despite variations in their relative abundance. This aligns with previous studies reporting limited trait loss in mesophotic compared to altiphotic communities (Bosch et al. 2023; Loiseau et al. 2023; Medeiros et al. 2021), but significant declines in rariphotic communities (Stefanoudis et al. 2023). In the upper section of reefs (0–120 m), we found high functional homogeneity across three taxonomically distinct fish communities: the altiphotic, upper mesophotic, and lower mesophotic. This core of shared ecological traits among taxonomically distinct reef-fish communities is also a common feature across latitudes (McLean et al. 2021; Mouillot et al. 2014) and likely reflects the ubiquity of essential ecological roles on reefs. However, many ecological roles on mesophotic reefs were supported by lower abundances and number of species, and often by depth generalists or by species occurring at the limit of their depth range. Previous studies have also found drops in over-redundancy, i.e., high concentrations of species in given functional entities, between altiphotic and mesophotic communities (Stefanoudis et al. 2025). Whether trait combinations supported by rare species occurrence still support ecologically meaningful functions on deep reefs is unclear (e.g., nutrient cycling, bioerosion, predator-prey interactions).

Hierarchical branching events indicated a functional break at the mesophotic/rariphotic boundary and between upper and lower rariphotic communities, and matched taxonomic breaks. These functional breaks suggest that while rariphotic communities are primarily composed of reef-associated families, they may fundamentally differ from shallower reefs in the variety and magnitude of ecological processes (Brandl et al. 2019). Lastly, the functional break and the sharp shrinkage of the trait space between rariphotic and below-rariphotic depths suggests a strong ecological shift below the rariphotic (300 m), in line with the taxonomic transition from reef-associated to deep-sea associated families occurring at this depth (Baldwin et al. 2018; Jacquemont et al. 2024a; Quattrini et al. 2017; Weijerman et al. 2019). This transition remains to be documented in other ocean basins.

### Environmental drivers of trait loss

Previous functional characterizations of deep-reef fishes have mostly focused on trophic guilds with an emphasis on the decline in herbivores with depth (Bridge et al. 2016; Loiseau et al. 2023; Richardson et al. 2023; Stefanoudis et al. 2023). While we also observed this trend, we found that additional trophic guilds, as well as behavioral and life-history traits, disappeared below the mesophotic. Indeed, we found that detritivores, ectoparasite cleaners, and sessile invertivores were absent in the rariphotic, likely due to the decreased coverage of associated food sources, such as corals, gorgonians, and sponges. Similarly, fewer detritus due to lower overall biomass on deep reefs could limit the presence of detritivores. Additional factors at play could include the decrease in fish density and the loss of visual cues from low light intensity, which are both required to support ectoparasite cleaning (Caves 2021).

The increase in benthic sedentary fishes on deep reefs could result from the decrease in habitat availability. On lower mesophotic and rariphotic reefs surveyed, habitats were characterized by isolated biotic and abiotic features among vast expanses of sand and rubble. In such environments where shelters are scarce and exposure to predation is high, territorial behaviors could have replaced roving demersal behaviors found on shallow reefs. Deep-reef fishes were often observed in tight association with specific benthic features. Monospecific (e.g., *Symphysanodon spp.*) or intergeneric (e.g., pairings of *Antigonia capro*s and *Ostichthys trachypoma*) groups often aggregated around large benthic features (e.g., rocky outcrop, sunken boat), and single territorial individuals (e.g., *Serranus spp.*) were observed living in association with a whip coral, a rock, or human trash (authors’ observations). The decrease in habitat complexity, microhabitat variability, and habitat-forming species on deep-reefs likely contributes to the decrease in species and trait richness through a loss of specialization opportunities (Costello and Chaudhary 2017).

### Mobility and reproduction traits shape deep-reef connectivity

We observed a decrease in the proportion of fishes displaying vertical and horizontal mobility with depth, which could result in lower population connectivity and nutrient cycling (Papastamatiou et al. 2015; Slattery et al. 2011). In the mesophotic, abundant planktivorous schooling species that perform diel vertical migrations, such as *Azurina spp.*, likely play an important role in maintaining nutrient connectivity with shallow reefs. In the rariphotic, we only observed a few large predators such as *Caranx spp.*, *Seriola spp.*, and *Pterois volitans* known to perform vertical migrations, but their low abundance might limit their capacity to move nutrients vertically or horizontally. Although potentially limited by low adult mobility, population connectivity on deep reefs could be supported by the higher proportion of reproductive strategies based on pelagic eggs, which enhances the dispersal capacity of species compared to benthic eggs. Additional factors, including environmental stability and geomorphological variability (Copus et al. 2022), determine rates of speciation and endemism on deep reefs, which remain highly debated (Jacquemont et al. 2024a; Kane et al. 2014; Pyle et al. 2016).

The low species richness and abundance of mobile species observed on deep reefs could either reflect (1) their actual ecological preference for shallow reef habitats, (2) their regional depletion across depth due to overfishing, (3) survey biases. Indeed, most highly mobile species observed on deep reefs were large predators that are primary targets of local fisheries, and known to be overfished (Barremore et al. 2021, 2023). Our survey method could also have led to the underestimation of pelagic vertical migrators since the submersible was always facing the reef slope. Further, some fast-swimming vertical migrators and large predators, such as sharks, which have been previously recorded down to 300 m on Caribbean reefs (Chapman et al. 2007), might have avoided the submersible during surveys. As such, the frequency and ecological role played by large mobile species on deep reefs remains uncertain and calls for complementary experimental approaches, such as baited video recordings or tagging surveys.

### The role of environmental filtering on deep reefs

In extreme environments such as deep ecosystems, functional diversity tends to be predominantly determined by abiotic constraints, a process referred to as environmental filtering (Myers et al. 2021). Communities under high environmental filtering typically display lower functional richness, lower functional divergence, and narrower trait space, while opposite trends denote the influence of high interspecific competition. We found decreases in functional richness, functional dispersion, and trait space with depth, indicating that the influence of environmental filtering increased with depth. The convergent trait filtering of species across phylogenetically distant families on deep reefs and the intrageneric diversification of some deep-reef specialists (Baldwin and Robertson 2014; Tornabene et al. 2016) also support that deep reefs favor specific trait combinations, another indication of strong environmental filtering. However, depth partitioning amongst congeners (Jacquemont et al. 2024a) indicates that resource limitations might prevent the coexistence of functionally similar species at a given depth zone, an indication of interspecific competition. Overall, findings support that environmental filtering combined with intrageneric diversification constitute strong evolutionary drivers on deep reefs (Bridge et al. 2016; Pinheiro et al. 2023a). At mesophotic depths, these trends were not as pronounced, which could explain why studies limited to the upper section of deep reefs find more ambiguous results [e.g., Medeiros et al. (2021)].

### From trait composition to vulnerability assessments of deep reefs

While we expected to observe a decrease in trait redundancy with depth due to species loss, we found that trait redundancy was stable down to the mesophotic and even increased in the rariphotic because species loss was concentrated on specific trait combinations. This process was also apparent from the increase in functional evenness with depth. While Stefanoudis et al. (2025) also found increases in functional evenness with depth and similar functional redundancy across altiphotic and upper mesophotic depths, both Stefanoudis et al. (2025); Pinheiro et al. (2023a) reported sharp decreases in redundancy in the lower mesophotic and below. This difference could stem from differences in the geographic areas studied (Indian Ocean and Atlantic reefs vs. Caribbean reef), in the survey methods employed, or in the metrics used to compute functional redundancy. Functional redundancy is thought to enhance community resilience because ecological roles supported by more species are likely to be conserved even when species are lost following a disturbance (MacArthur 1955; McLean et al. 2019). Under this “insurance hypothesis”, mesophotic and rariphotic communities could benefit from higher resilience thanks to higher trait redundancy. However, higher redundancy on deep reefs resulted from the loss of rare traits rather than from an increase in the number of species representing each trait. Similarly, higher evenness, which has also been linked to higher resistance (Oliver et al. 2015), resulted from a decrease in abundant species. Lowered abundance and trait diversity could undermine the resilience of deep reefs according to the “diversity-stability” hypothesis (Duffy et al. 2016; Isbell et al. 2015). Whether the trade-off between redundancy and diversity promotes resilience or vulnerability of deep reefs likely depends on additional factors, including trait composition and the type of pressures at play.

Trait-based approaches represent valuable tools to characterize ecological vulnerability, ecosystem processes, and evolutionary drivers, but they are now well known to quantify functional potential rather than actual ecological processes (Brandl et al. 2019). Further, the use of trait categories originating from shallow reef-fish ecology might fail to capture the full spectrum of deep-reef trait diversity. As deep-reef ecology progresses, a finer granularity in the niches occupied by species might reveal higher levels of niche partitioning and hence lower redundancy at scales that are biologically relevant. This, combined with a better understanding of the anthropogenic and environmental pressures at play on deep reefs (Pinheiro et al. 2023b; Jacquemont et al. 2024b), will allow to better inform the conservation and management of these ecosystems (Bell et al. 2022; Soares et al. 2020). In the meantime, the growing pool of evidence that deep-reef communities harbor unique taxonomic and functional diversity supports that conservation efforts should encompass the entire depth range of reef ecosystems (0–300 m) to achieve ecologically representative marine protection.

## Electronic Supplementary Material

Below is the link to the electronic supplementary material.

Supplementary Material 1

Supplementary Material 2

## Data Availability

Data are already published and publicly available, with those items properly cited in this submission. Datasets, including a compilation of all fish observations and the compilation of traits assigned to each species, as well as R scripts used to conduct data analyses and create figures are available on Zenodo under the DOI: 10.5281/zenodo.14908246.

## Code availability

The annotated history of all R scripts are available under the public Github repository of Juliette Jacquemont (https://github.com/jjacquemont/Deep.Reef.Fish.Carib*)*.
