# Supplementary material for: Depth is a strong driver of functional diversity in Caribbean reef-fish communities
Source: Oecologia. 2026 May 15;208(6):67. doi: 10.1007/s00442-026-05902-9 (PMC13179212; doi:10.1007/s00442-026-05902-9)
Supplement: Supplementary file 2 — Supplementary Material 2 [file 442_2026_5902_MOESM2_ESM.pdf]

Depth is a strong driver of functional diversity in Caribbean reef-fish communities

Supplementary material

Juliette Jacquemont [1,2*], Simon J. Brandl [3], Carole C. Baldwin [4], Luke Tornabene [1]

[1] School of Aquatic and Fishery Sciences, University of Washington, 1122 NE Boat St, Seattle, WA 98195, United States

[2] National Center for Ecological Analysis and Synthesis, University of California, 1021 Anacapa St, Suite 300, Santa Barbara, CA 93101, United States

[3] Department of Marine Science, The University of Texas at Austin, Marine Science Institute, 750 Channel View Dr, Port Aransas, TX 78373, United States

[4] Department of Vertebrate Zoology, National Museum of Natural History, Smithsonian Institution, Washington, DC, USA

*** Corresponding author:** Juliette Jacquemont. Email: juliette.jacquemont.fr@gmail.com.

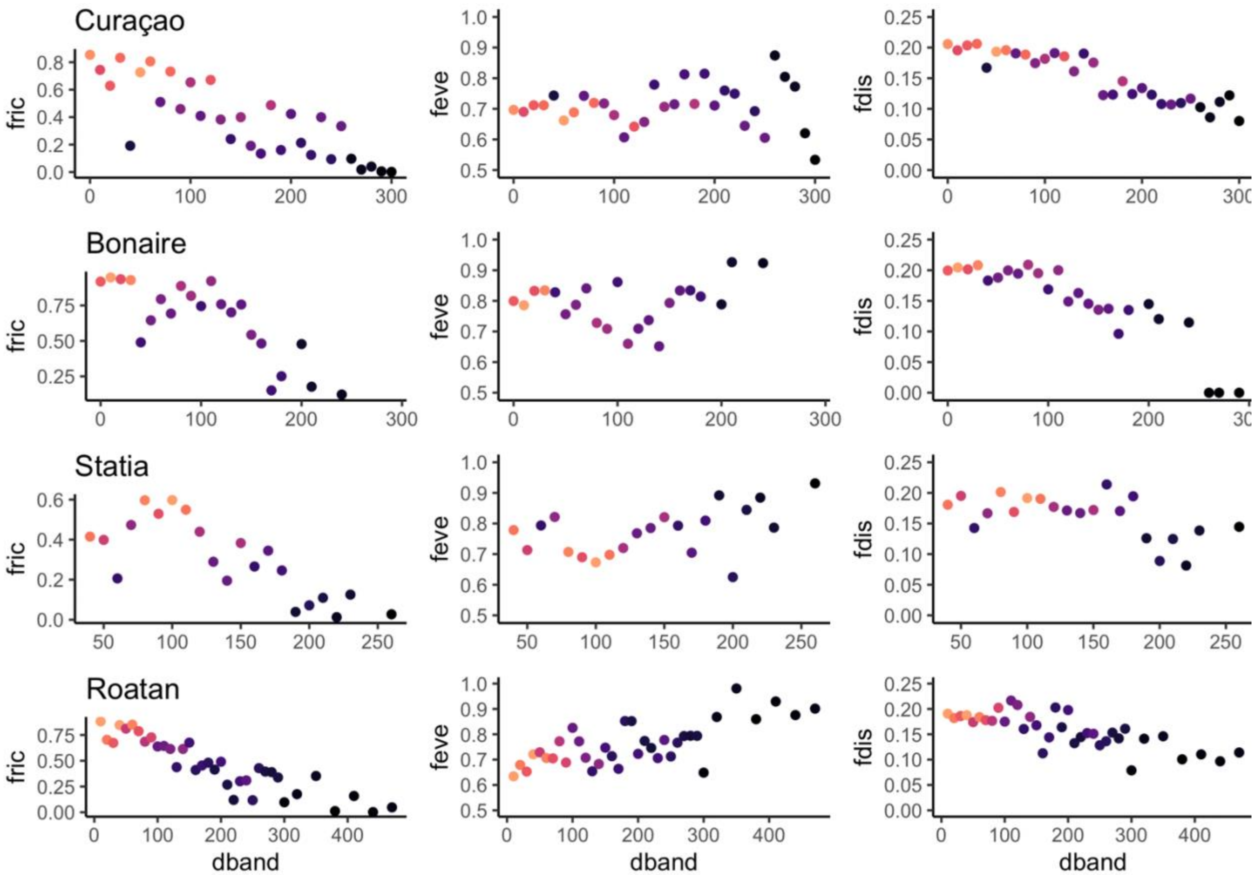

Fig. S1: Functional richness, evenness, and dispersion of reef-fish communities across depth at each of the four study sites. Colors denote species richness of communities

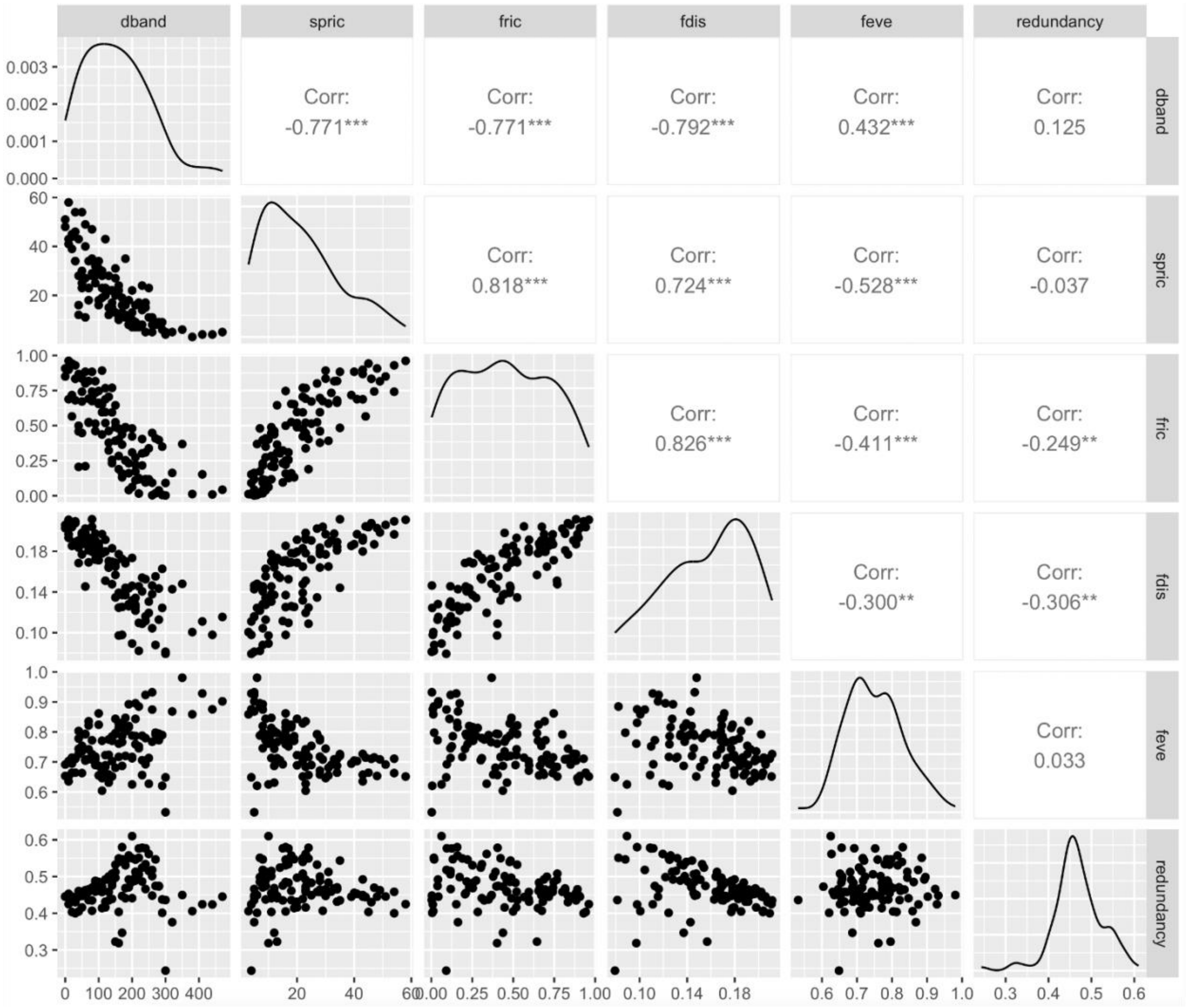

Fig. S2: Relations between depth (dband), species richness (spric), functional richness (fric), functional dispersion (fdis), functional evenness (feve), and functional redundancy

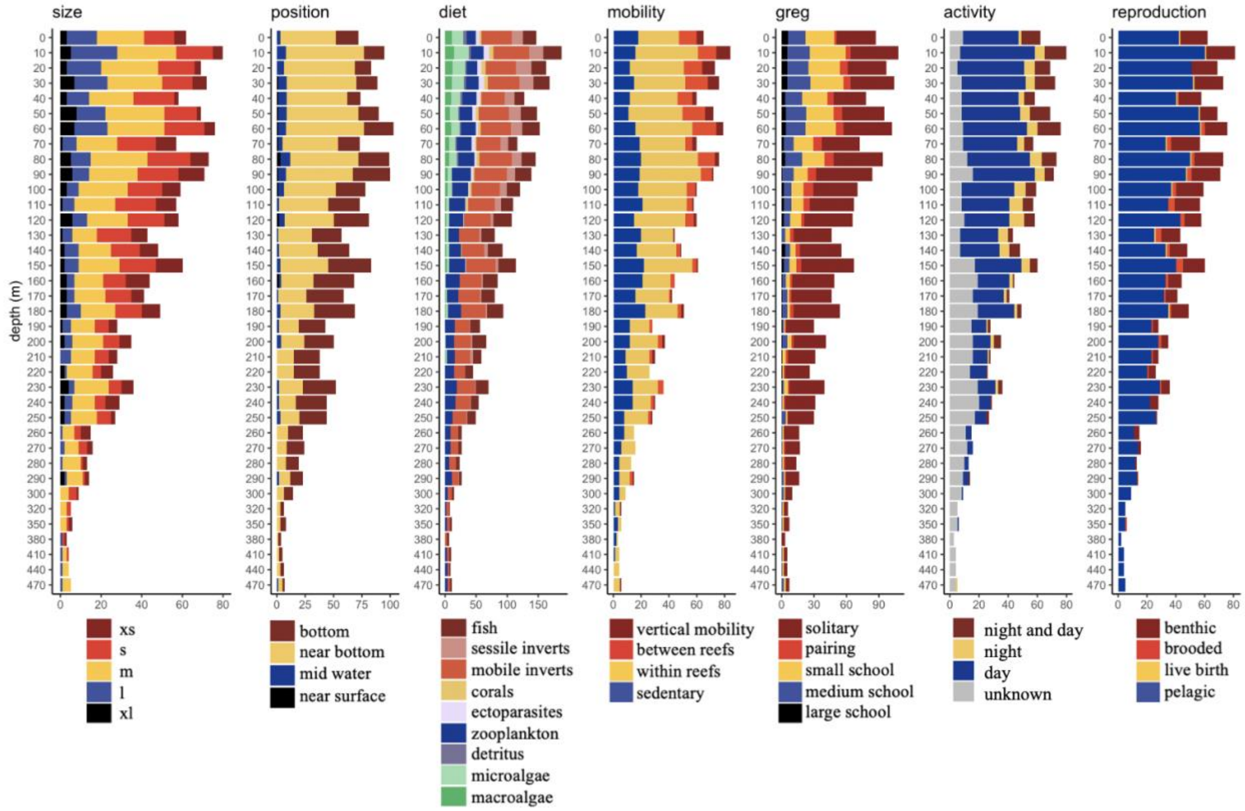

Fig. S3: Number of species representing trait categories across depth. Trait categories are represented by different colors and bar height represents species richness

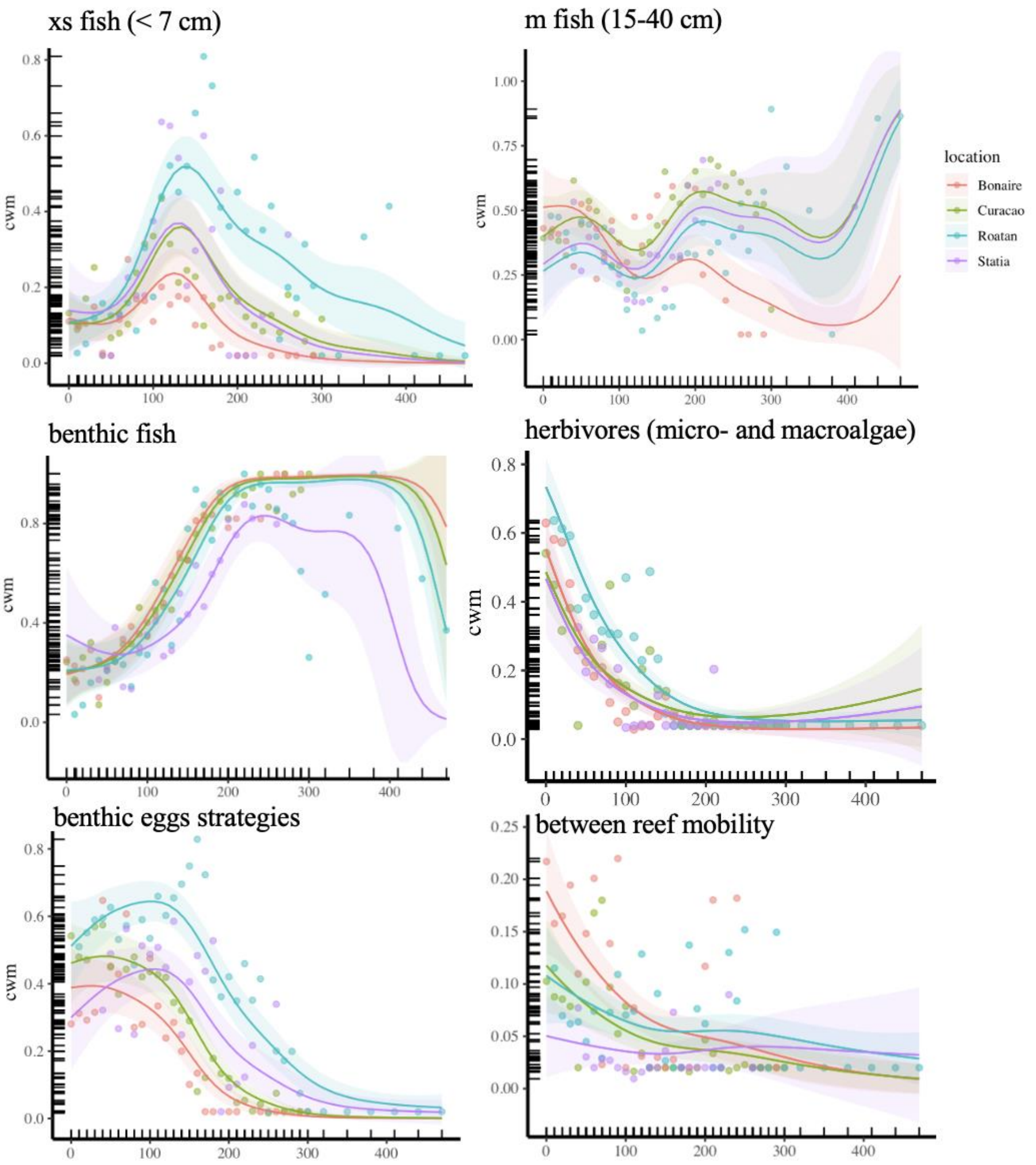

Fig. S4: Community weighted means of functional traits across depth at the four study locations. Data were fitted using General Additive Models with an interaction effect between depth and location. Shaded area depicts 95% confidence interval. Points represents observed values and colors indicate sites

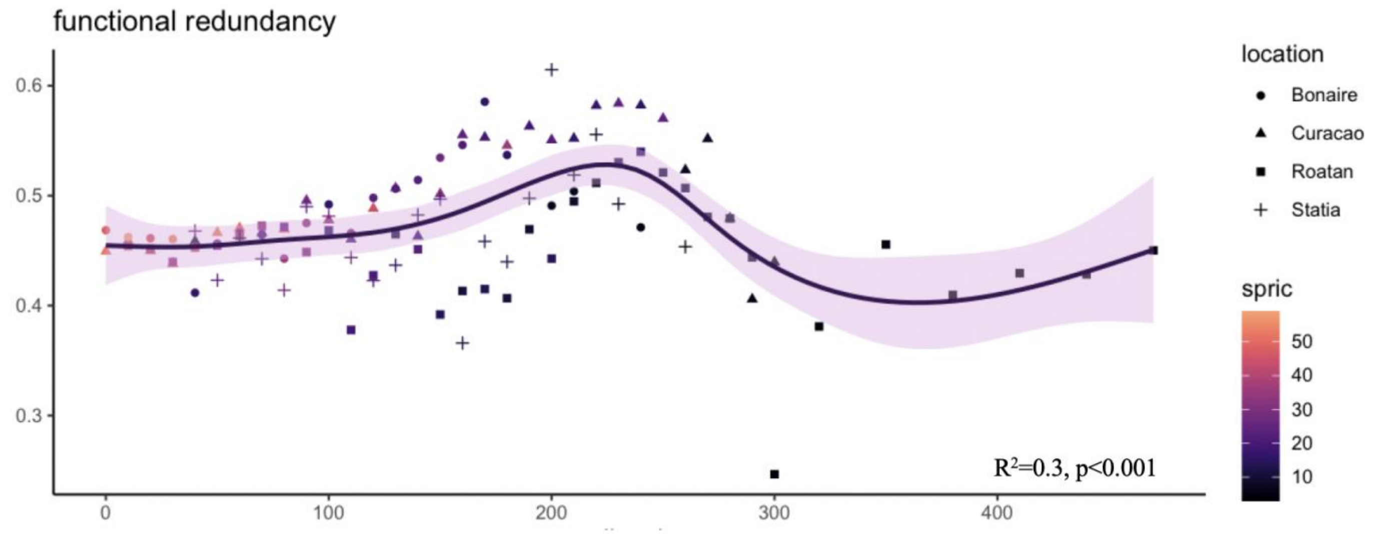

Fig. S5: Functional redundancy of fish communities across depth. Each dot represents a community from a given 10m depth bin at a given study site. Shapes of dots indicate the study site and color of dots indicate the species richness of the community. Dark lines represent the loess regressions fitted to the data and purple shading indicates the 95% confidence interval associated with the model. Residual standard errors (RSE) associated with the loess regressions are indicated in each panel

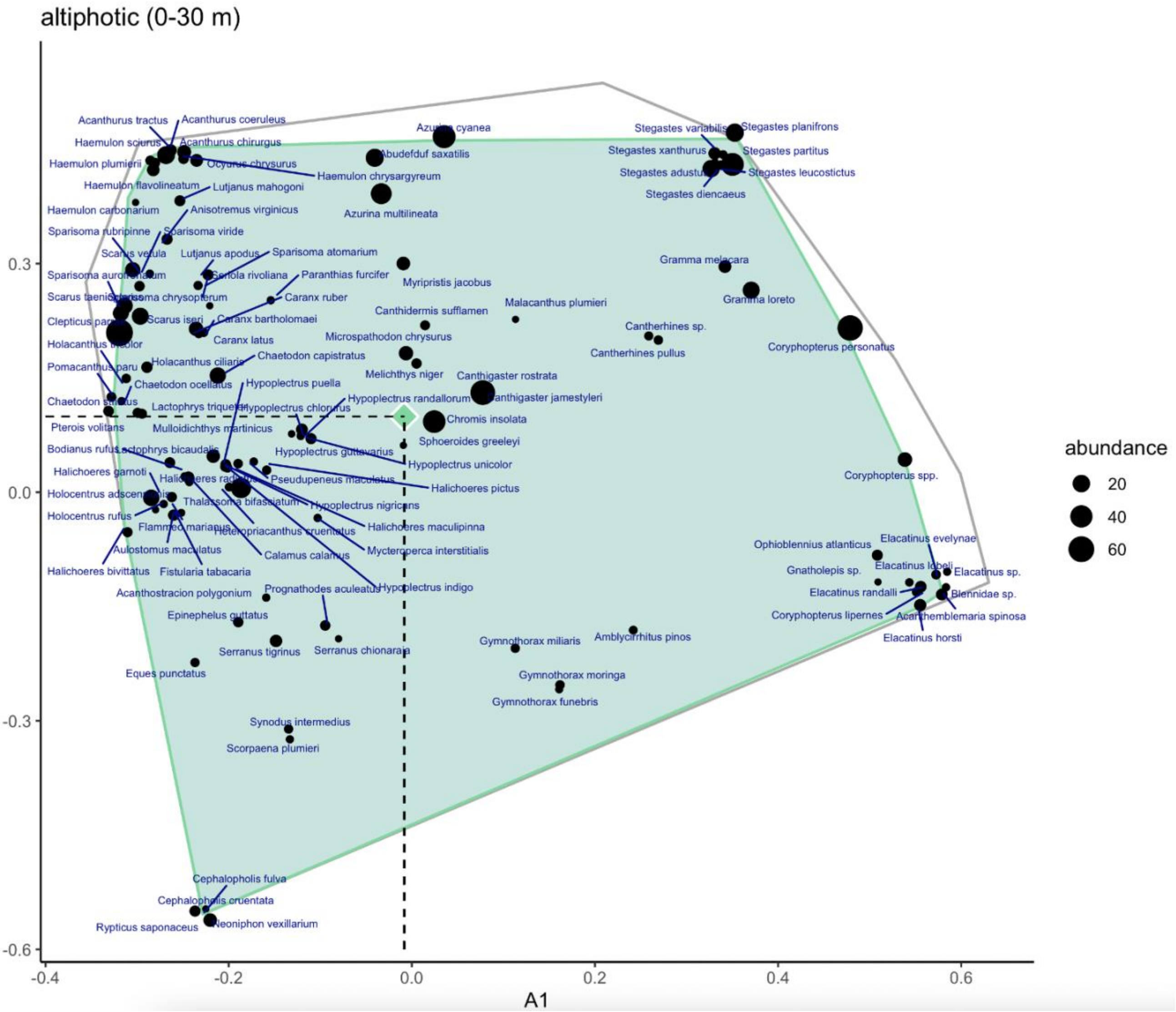

Fig. S6: Species composing the trait space of altiphotic communities

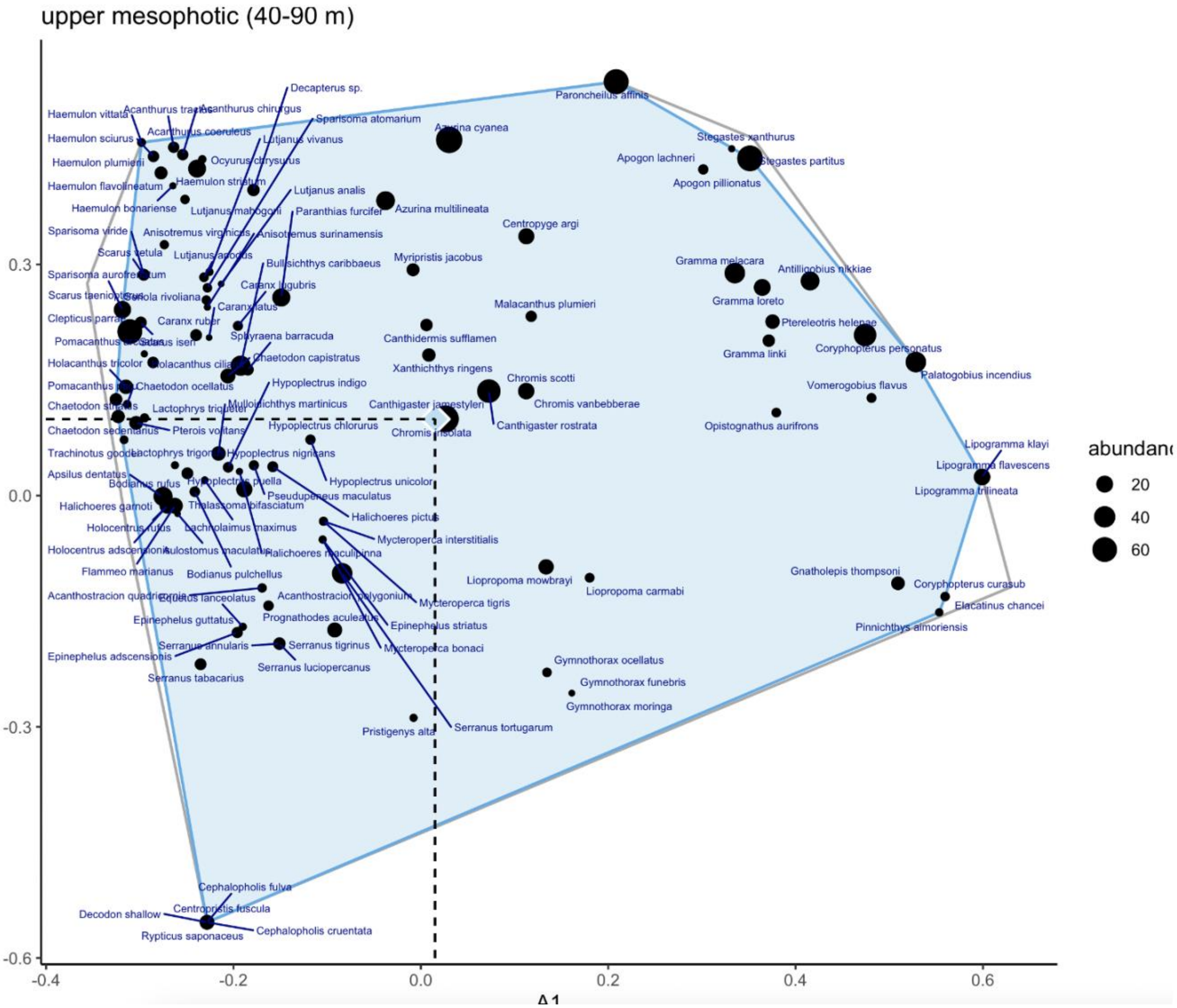

Fig. S7: Species composing the trait space of upper mesophotic communities

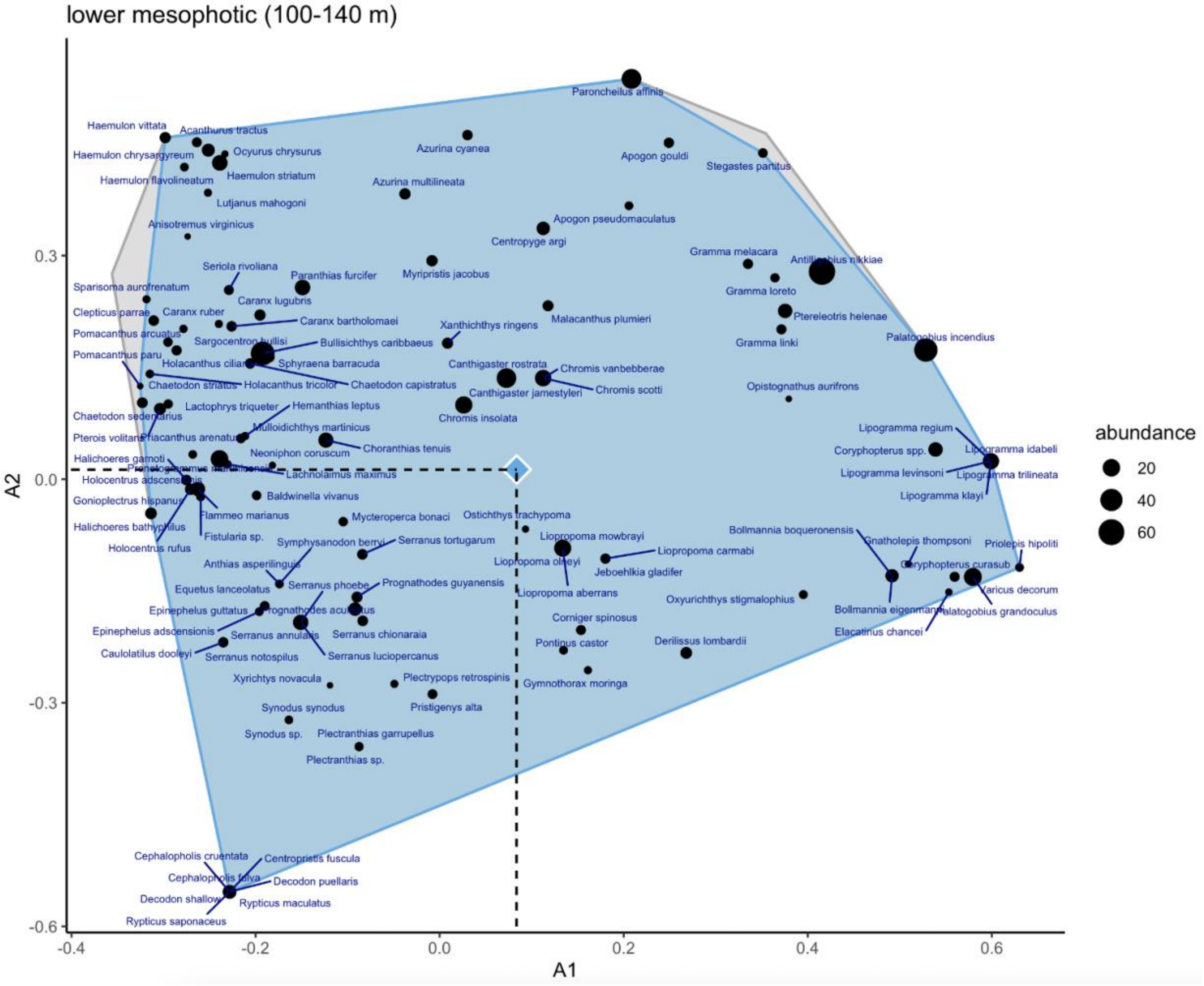

Fig. S8: Species composing the trait space of lower mesophotic communities

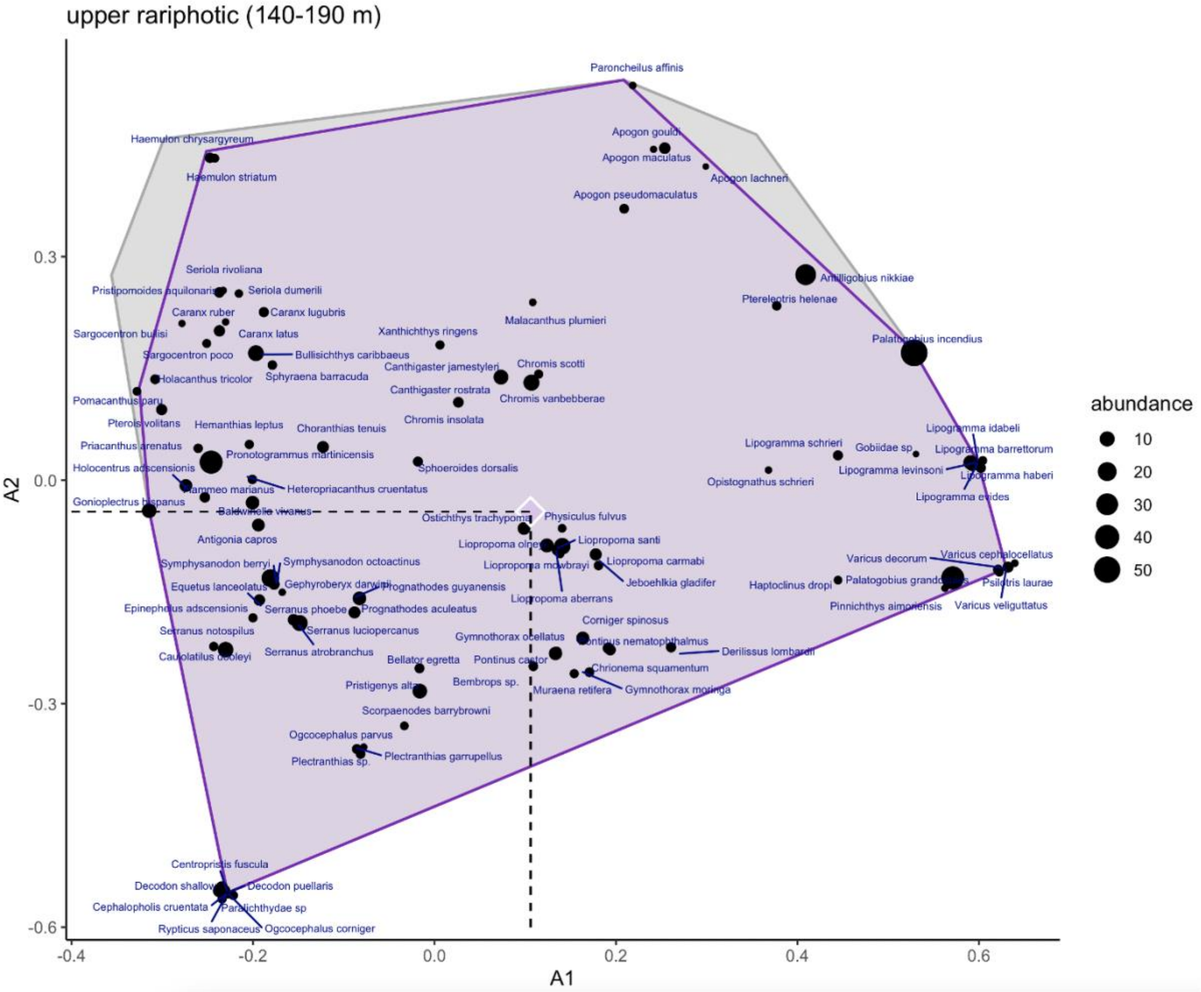

Fig. S9: Species composing the trait space of upper rariphotic communities

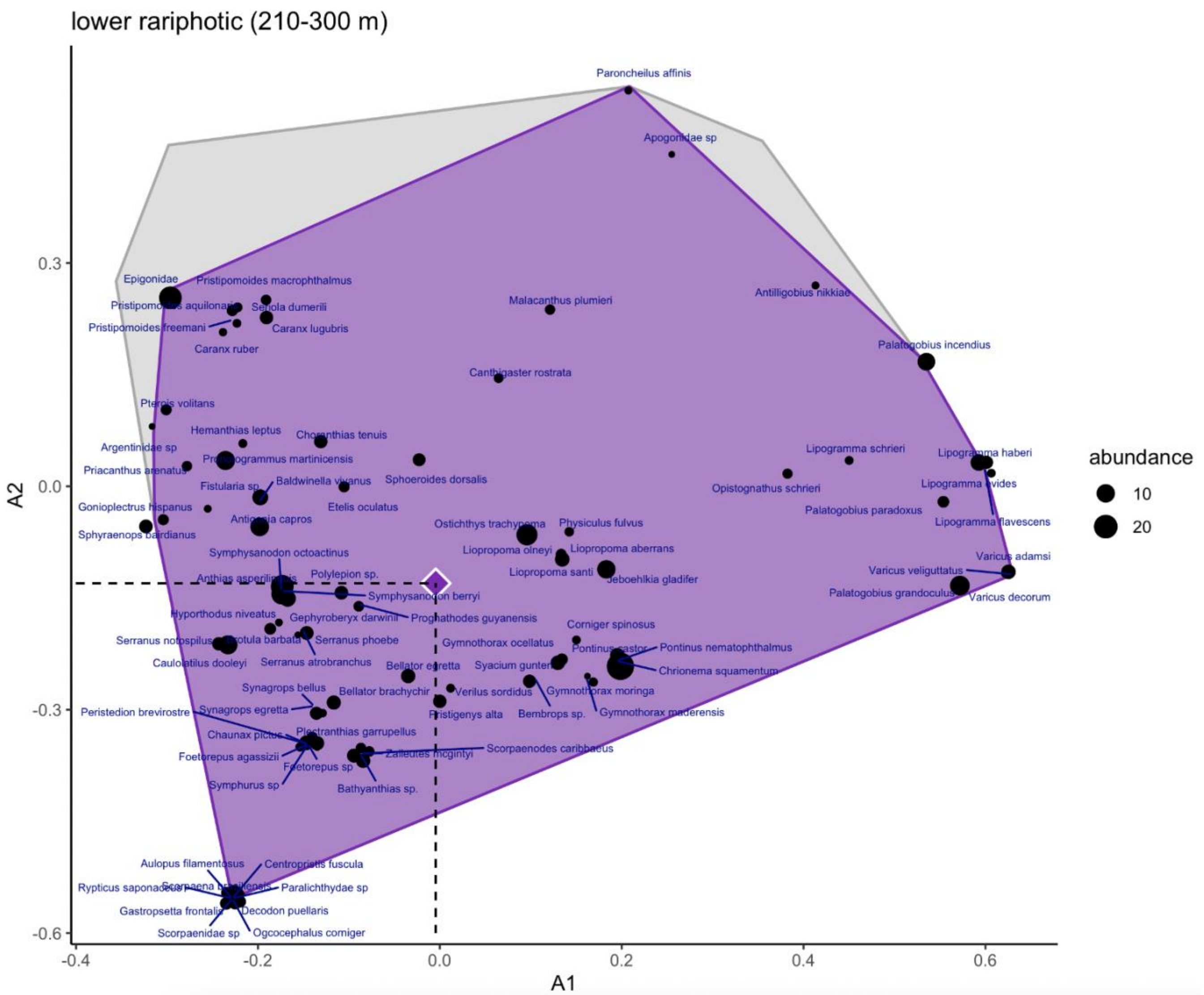

Fig. S10: Species composing the trait space of lower rariphotic communities

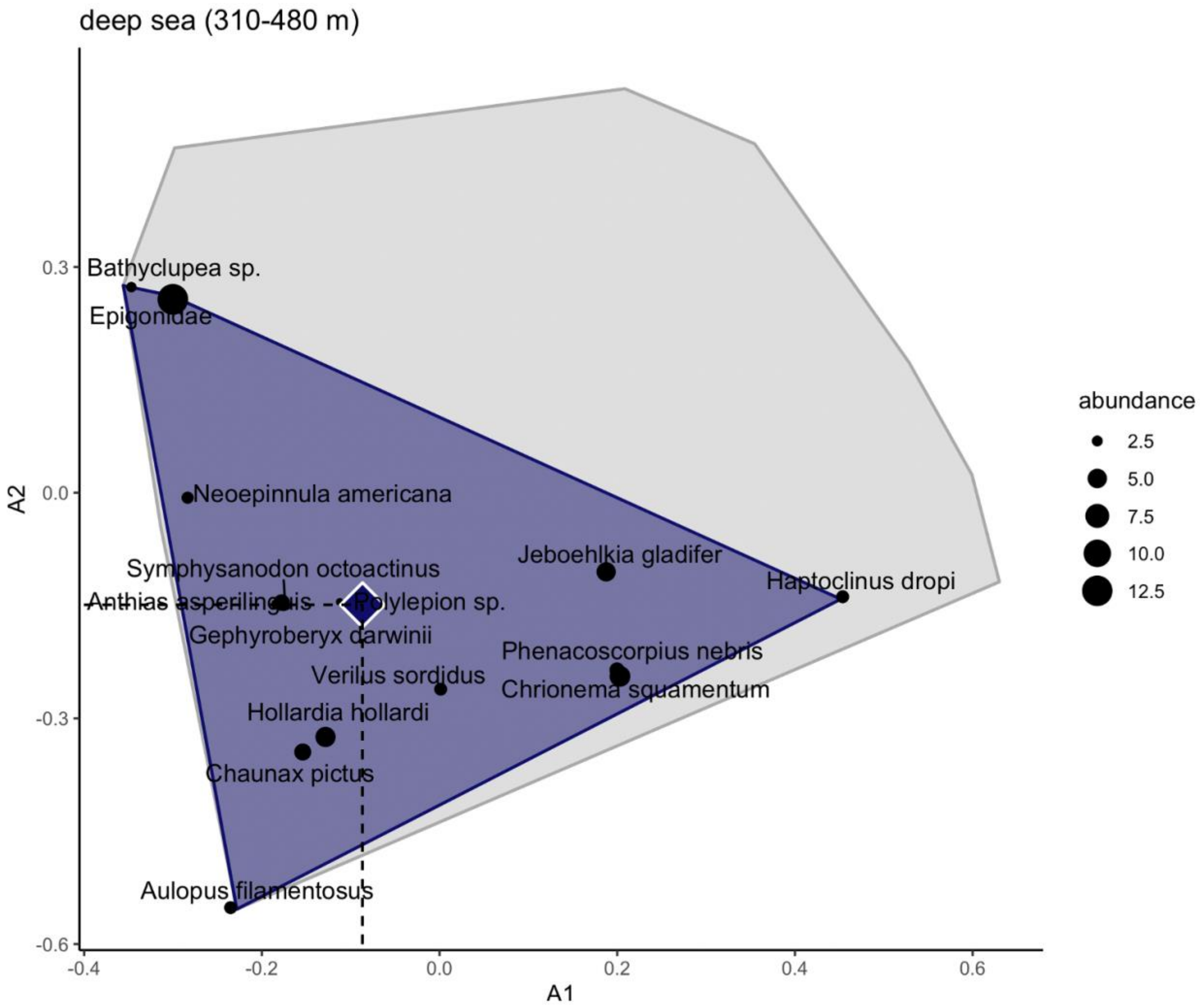

Fig. S11: Species composing the trait space of below-rariphotic communities

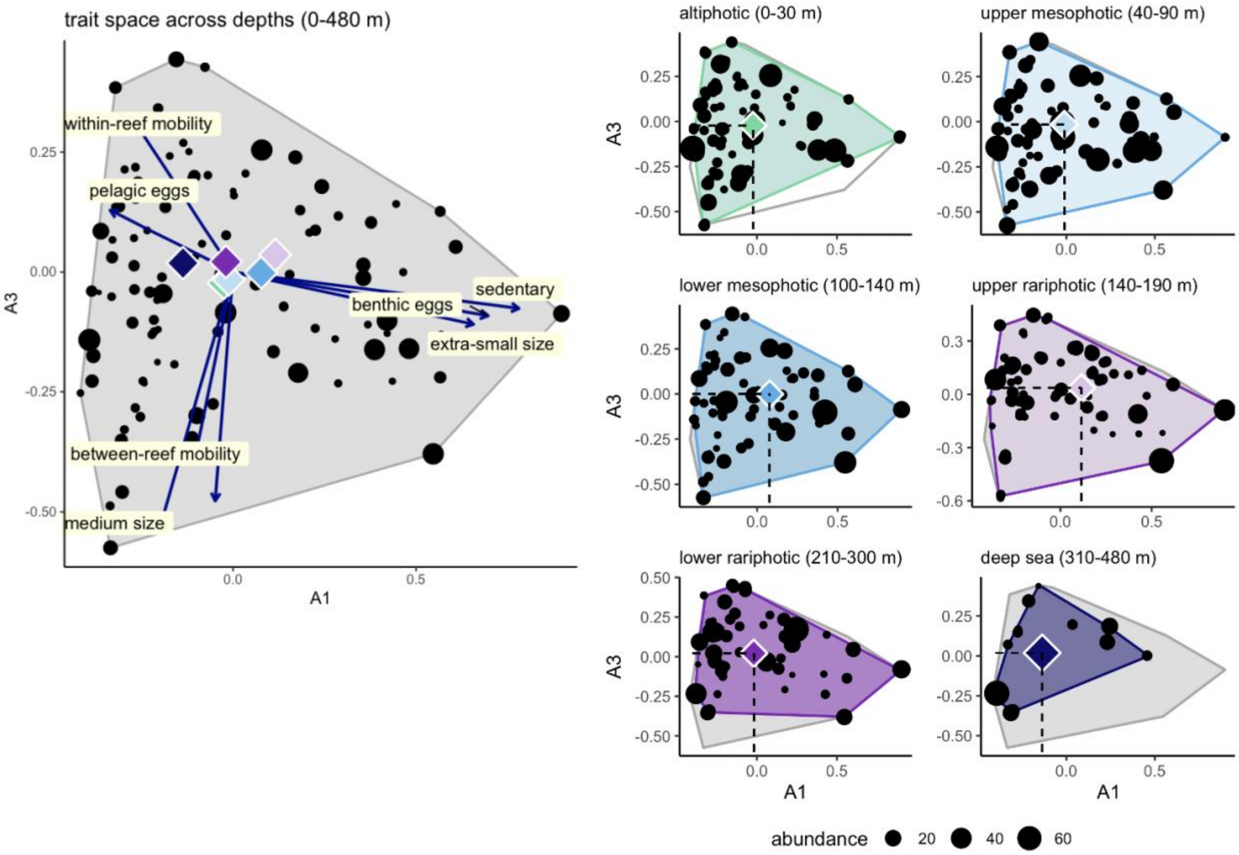

Fig. S12: Trait space occupied by fish communities across depths represented along the first and third Principal Coordinate Analyses (PCoA) axes. Black points represent species' position in the trait space and size of points represents the relative abundance of these species. Colored diamonds indicate the trait centroid for each depth zone. Vectors (left plot) represent the correlation of traits to the two principal component axes

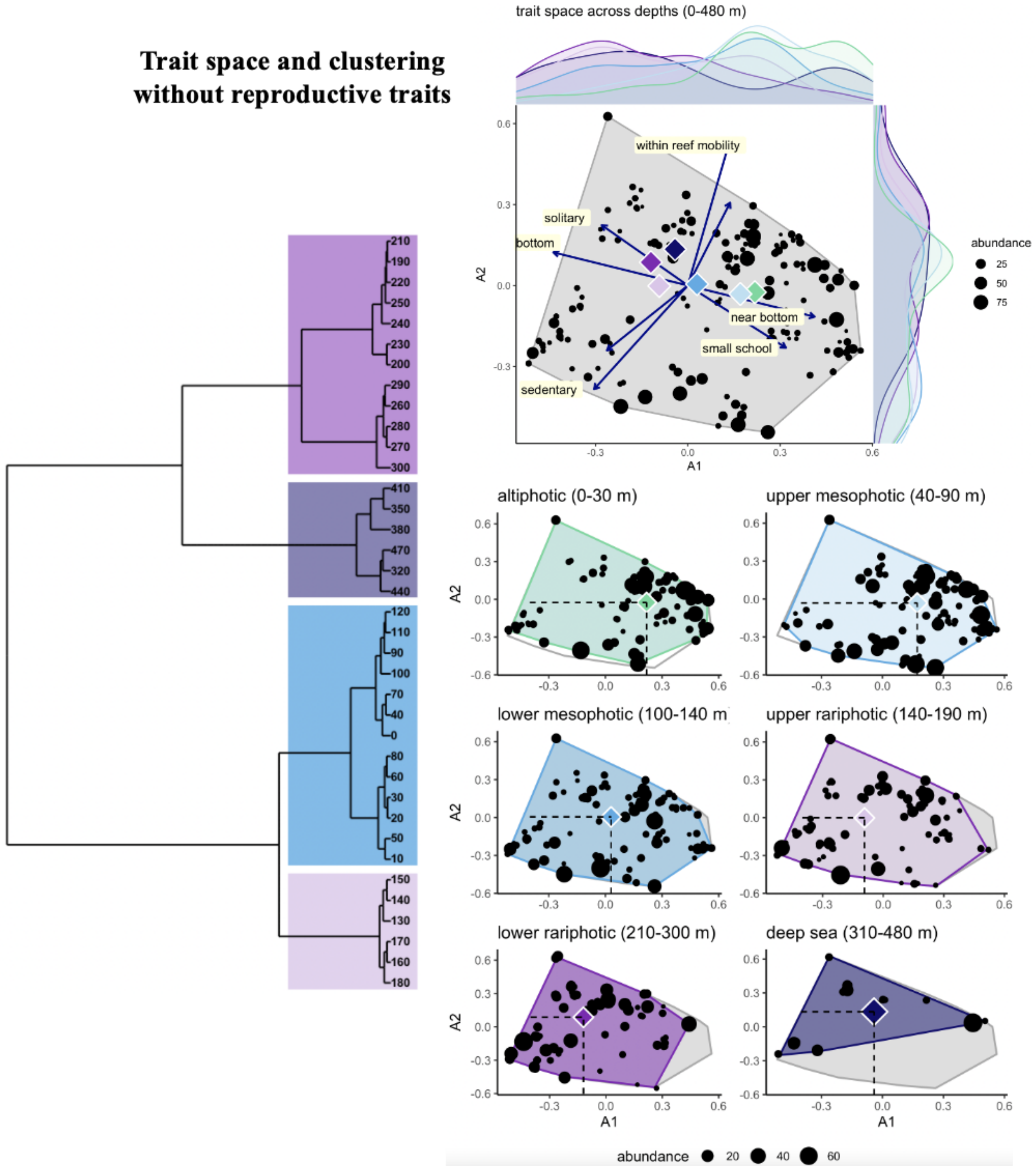

Fig. S13: Hierarchical clustering (right) and Principal Coordinates Analyses (PCoA) of fish communities across depths based on all traits but reproduction. Black points represent species' position in the trait space and size of points represents the relative abundance of these species. Colored diamonds indicate the trait centroid for each depth zone. Vectors (top plot) represent the correlation of traits to the PCoA axes

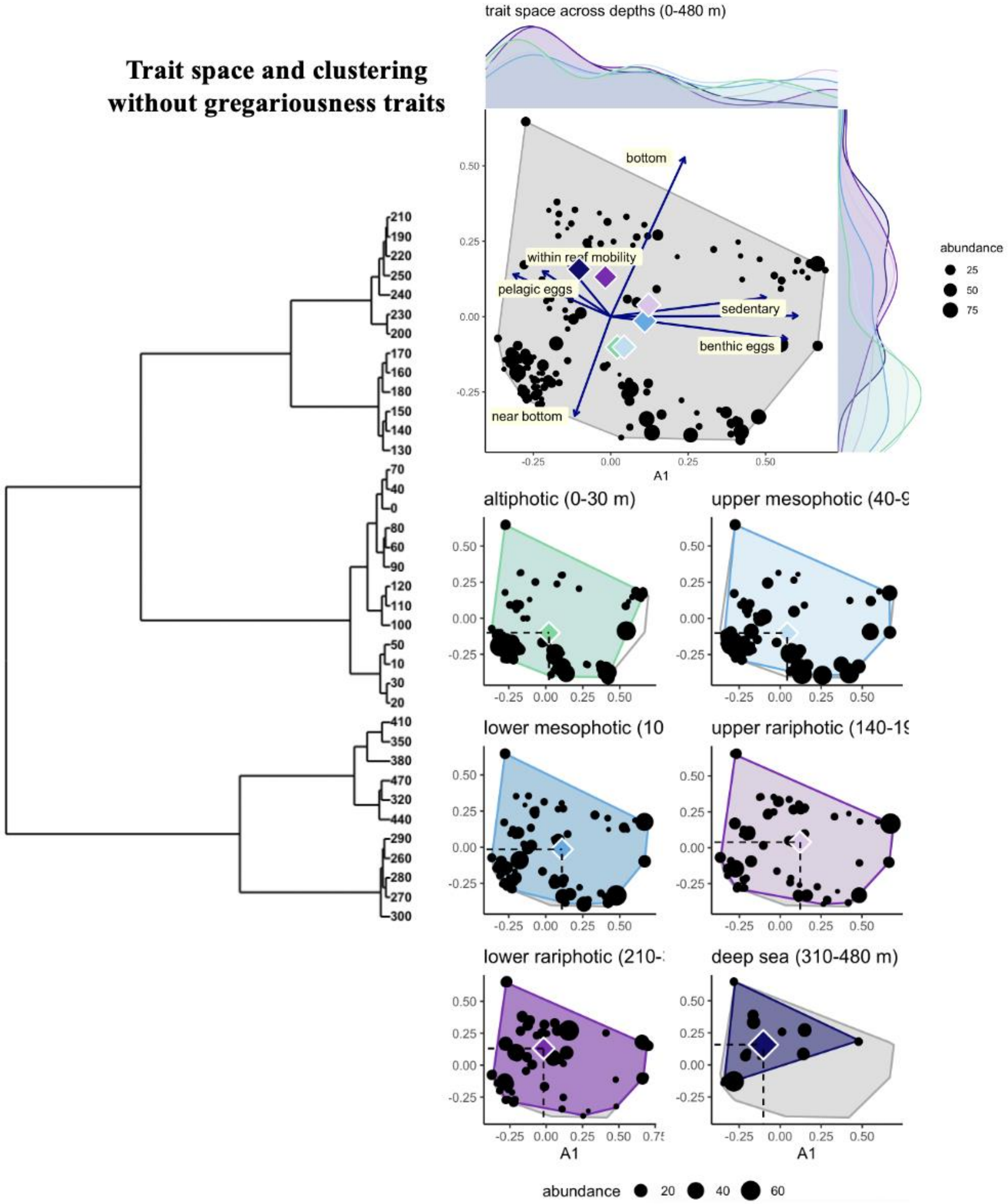

Fig. S14: Hierarchical clustering (right) and Principal Coordinates Analyses (PCoA) of fish communities across depths based on all traits but gregariousness. Black points represent species' position in the trait space and size of points represents the relative abundance of these species. Colored diamonds indicate the trait centroid for each depth zone. Vectors (top plot) represent the correlation of traits to the PCoA axes

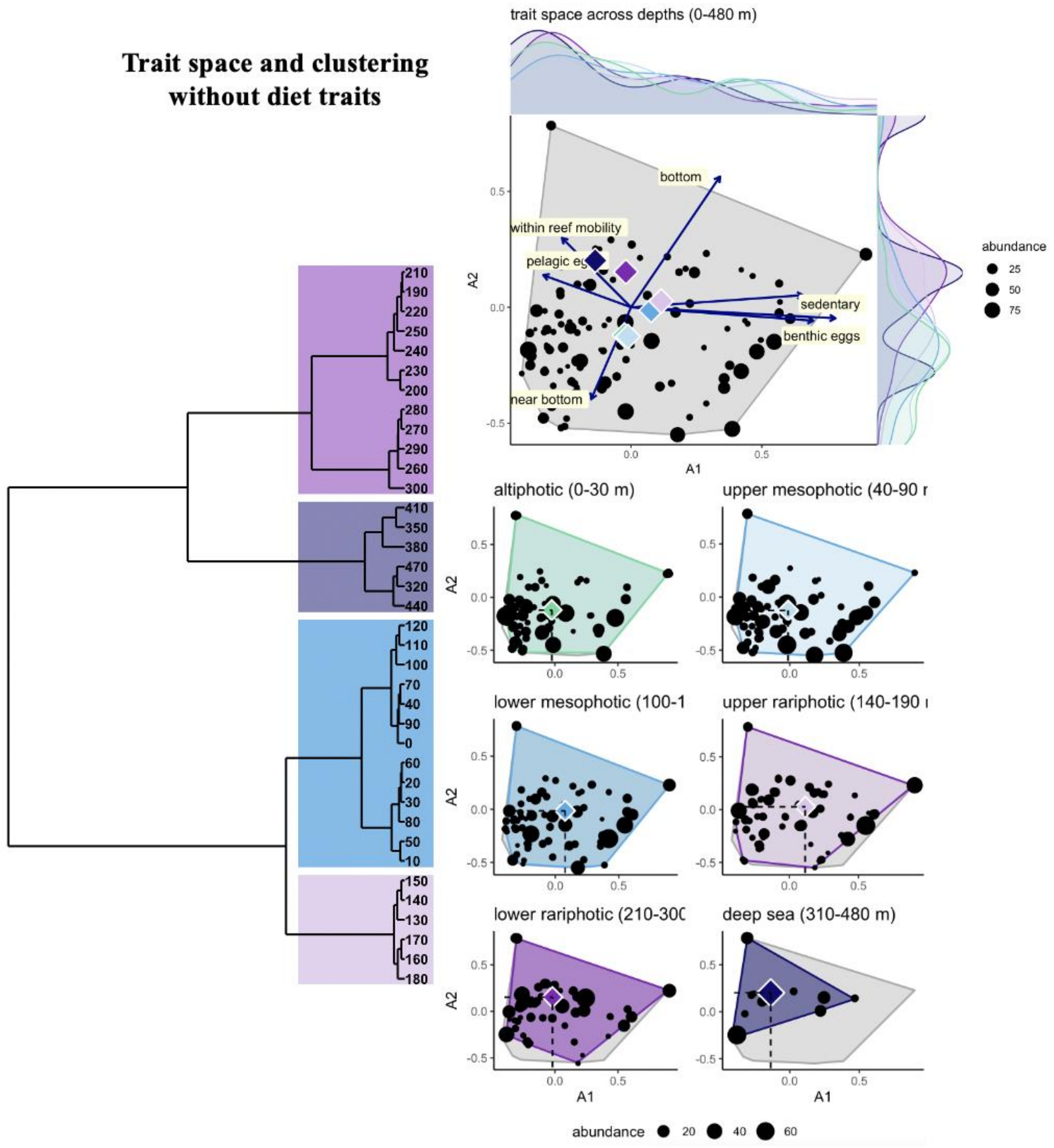

Fig. S15: Hierarchical clustering (right) and Principal Coordinates Analyses (PCoA) of fish communities across depths based on all traits but diets. Black points represent species' position in the trait space and size of points represents the relative abundance of these species. Colored diamonds indicate the trait centroid for each depth zone. Vectors (top plot) represent the correlation of traits to the PCoA axes

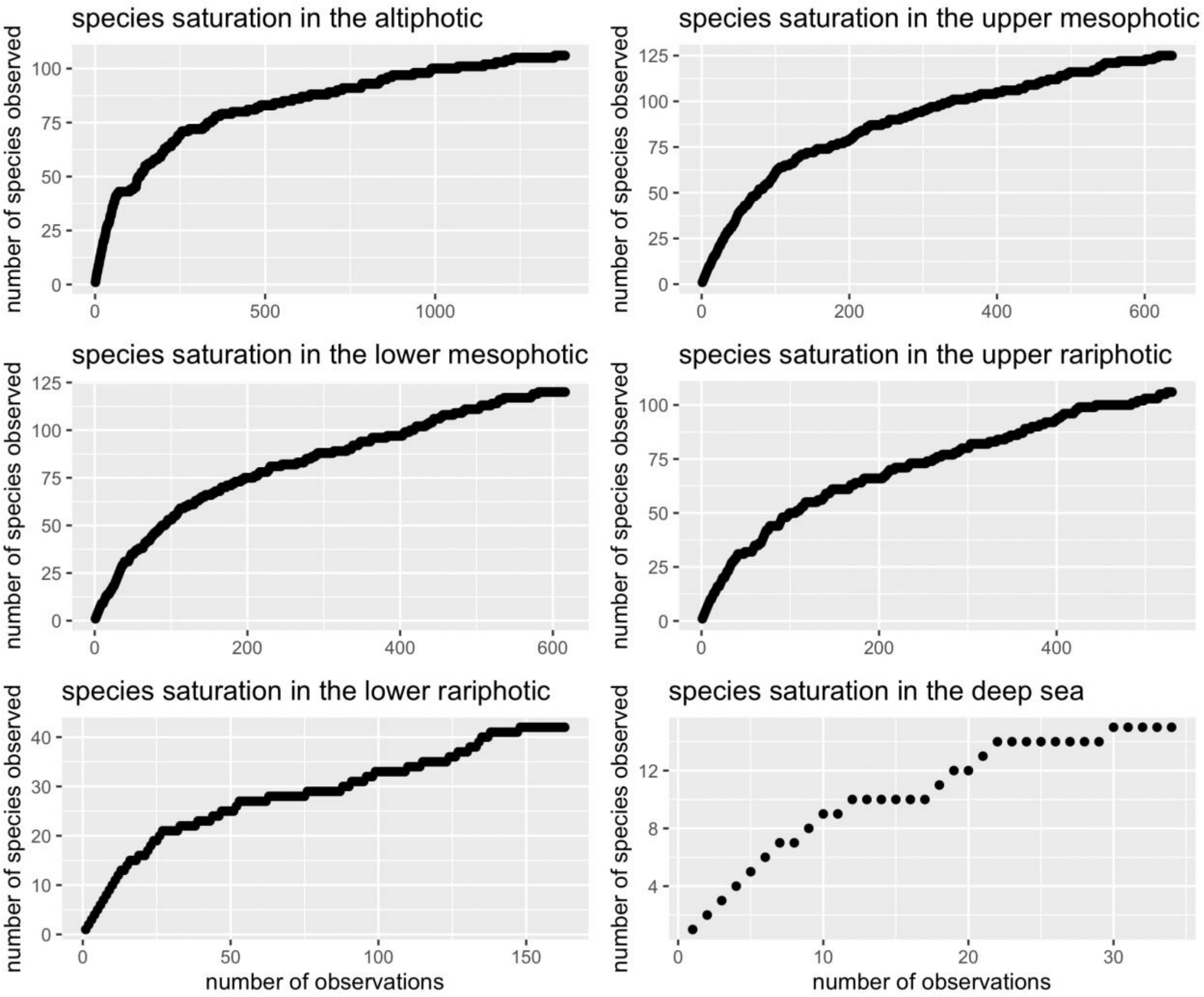

Fig. S16: Rarefaction curves for species observed at each depth strata

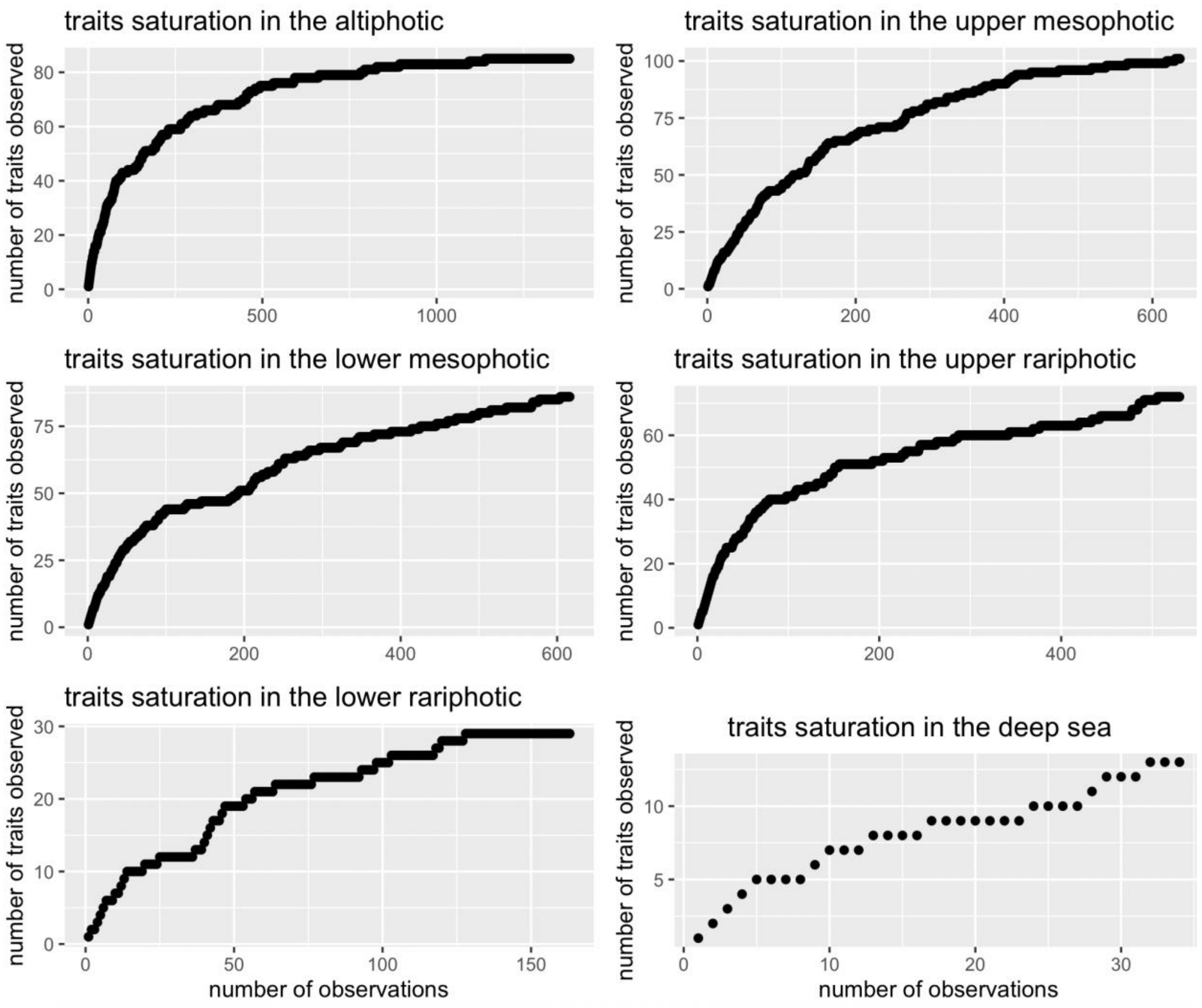

Fig. S17: Rarefaction curves for functional entities observed at each depth strata

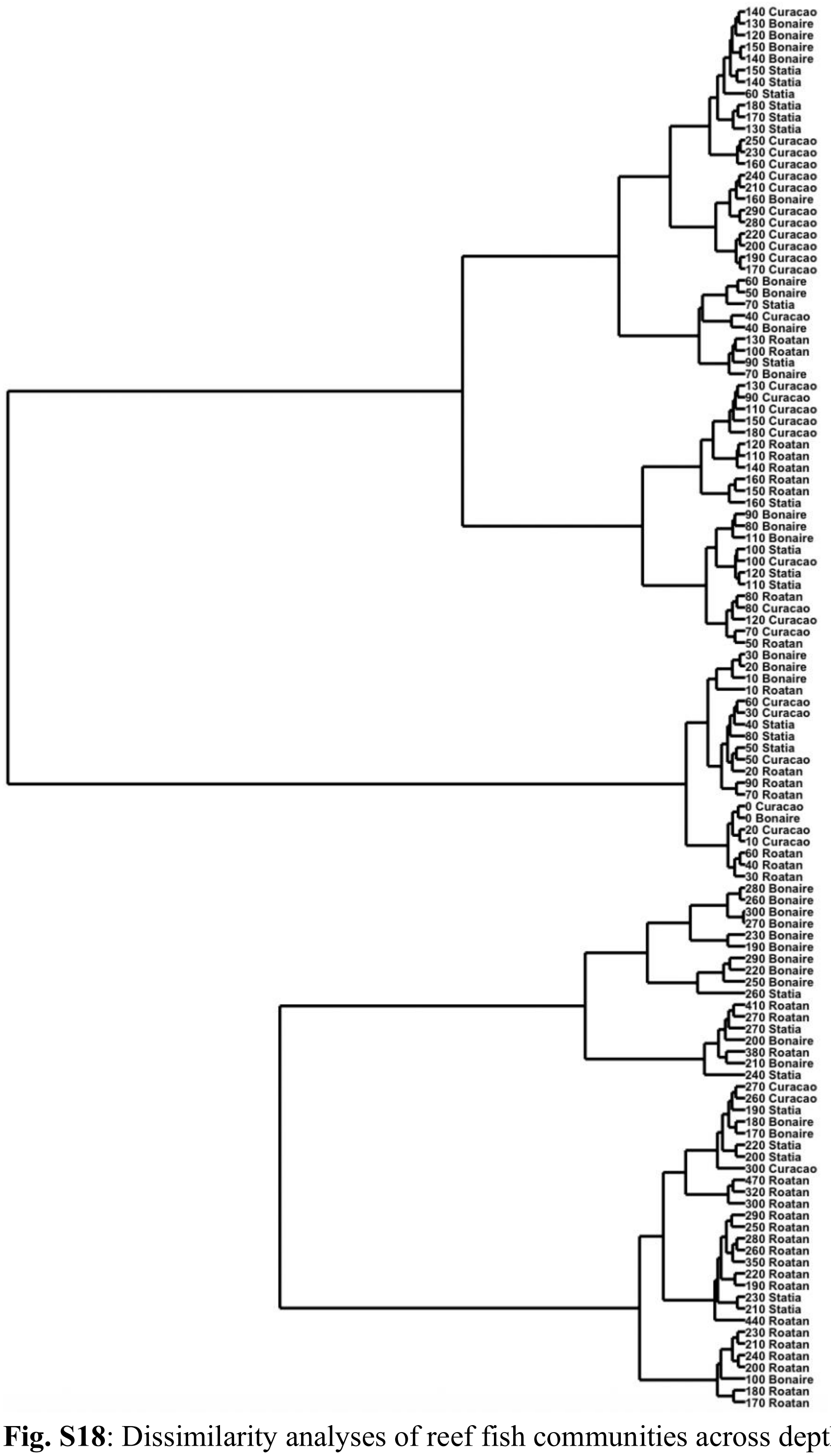

Fig. S18: Dissimilarity analyses of reef fish communities across depths based on trait structure. Length of branches in the dendrogram is commensurate to the dissimilarity between communities based on Bray–Curtis distance

Table S1. Number of transect replicates and total transect length (resp. time) surveyed by SCUBA (resp. submersible) at each site

**site**	**depth (m)**	**SCUBA transect replicates**	**total length (m)**
Curaçao	5-7	5	128
	10	5	126
	20	5	162
	30	6	124
Bonaire	5	6	180
	10	6	180
	20	6	180
	30	6	180

**site**	**depth (m)**	**submersible transect replicates**	**total time (hours)**
Curaçao	40-300	~ 100	~ 300
Bonaire	40-300	11	~ 40
Statia	40-300	12	~ 60
Roatan	10-470	17	~ 60

Table S2. Categories of traits considered to characterize fish species.

diet	water column position	reproduction	size at maturity	diel activity	gregariousness	mobility
macroalgae	bottom	benthic eggs	< 7 cm: 'extra small'	nocturnal	solitary	sedentary
microalgae	near bottom	brooding	7-15 cm: 'small'	diurnal	pairing	mobile within reef
bony fish	mid-water	pelagic eggs	15-40 cm: 'medium'	both	small school (3-20)	mobile between reefs
detritivore	near surface	livebearer	40-80 cm: 'large'		medium school (20-50)	vertical mobility
ectoparasite			> 80 cm: 'extra large'		large school (>50)	
cleaner						
corallivore						
mobile invertebrates						
sessile invertebrates						
planktivore						
